# The pluralization palette: unveiling semantic clusters in English nominal pluralization through distributional semantics

**DOI:** 10.1007/s11525-024-09428-9

**Published:** 2024-07-12

**Authors:** Elnaz Shafaei-Bajestan, Masoumeh Moradipour-Tari, Peter Uhrig, R. Harald Baayen

**Affiliations:** 1 Department of General and Computational Linguistics, University of Tübingen, Wilhelmstraße 19, Tübingen, 72074 Baden-Württemberg Germany; 2 Department of English and American Studies, Friedrich-Alexander-Universität Erlangen-Nürnberg, Bismarckstraße 1, Erlangen, 91054 Bayern Germany

**Keywords:** Pluralization, Plural semantics, Distributional semantics, Word embeddings, Proportional analogies, Vector averaging, Compositional distributional semantics

## Abstract

Using distributional semantics, we show that English nominal pluralization exhibits semantic clusters. For instance, the change in semantic space from singulars to plurals differs depending on whether a word denotes, e.g., a fruit, or an animal. Languages with extensive noun classes such as Swahili and Kiowa distinguish between these kind of words in their morphology. In English, even though not marked morphologically, plural semantics actually also varies by semantic class. A semantically informed method, CosClassAvg, is introduced that is compared to two other methods, one implementing a fixed shift from singular to plural, and one creating plural vectors from singular vectors using a linear mapping (FRACSS). Compared to FRACSS, CosClassAvg predicted plural vectors that were more similar to the corpus-extracted plural vectors in terms of vector length, but somewhat less similar in terms of orientation. Both FRACSS and CosClassAvg outperform the method using a fixed shift vector to create plural vectors, which does not do justice to the intricacies of English plural semantics. A computational modeling study revealed that the observed difference between the plural semantics generated by these three methods carries over to how well a computational model of the listener can understand previously unencountered plural forms. Among all methods, CosClassAvg provides a good balance for the trade-off between productivity (being able to understand novel plural forms) and faithfulness to corpus-extracted plural vectors (i.e., understanding the particulars of the meaning of a given plural form).

**Supplementary Information:**

The online version contains supplementary material available at 10.1007/s11525-024-09428-9.

## Introduction

In his introduction to theoretical linguistics, Lyons (1968) states that “On the basis of a proportion like *boy : boys*, we can form ‘analogically’ thousands of other words: *cow : cows, girl : girls*, etc.; given either *cow* or *cows*, we can ‘solve’ the equation *boy : boys* = *cow : x* or *boy : boys* = *x : cows*.” (p. 6) (see also Haspelmath & Sims, 2010; Booij, 2010). In this proportional analogy, the semantics of pluralization is assumed to be the same across all regular nouns.

The proportional analogy for English noun singulars and plurals can be narrowed down in different ways. The opposition between singulars and plurals can be taken to be privative, 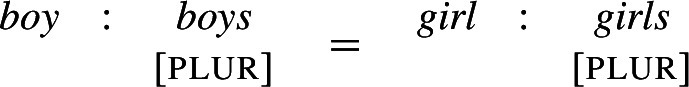 but also as equipollent, 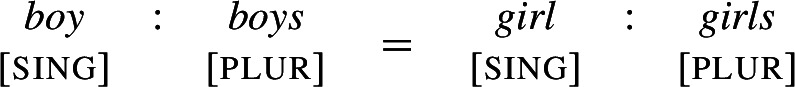
(see, e.g. Stump, 2019). Various proposals have been put forward addressing in what way plurals differ in meaning from their corresponding singulars. From the vast literature on this issue, we highlight only a few examples. According to Lasersohn (1995), plurality just means ‘more than one’. Link (1983/2012), in his logical analysis of plurals, takes noun singulars to be atoms and noun plurals to be non-atomic sums of atoms. de Swart and Farkas (2010) pointed out that plurals can be used in a more generic sense to denote one or more atoms, as in

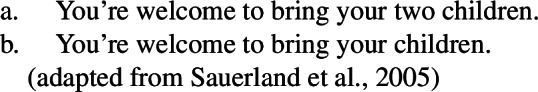

In (1-b), but not in (1-a), *children* can mean ‘one’ child or ‘more than one’ children. (Heim, 1991/2008), Sauerland et al. (2005) and Liter et al. (2017) argue that whether a plural form is understood as denoting multiple instances is a consequence of pragmatic inference, and highly context-dependent (see also Chemla, 2008). It is also possible for plurals to be unspecified with respect to number, a use that Mattens (1970) refers to as the *indifferentialis*. All these interpretations of noun pluralization have in common the assumption that the semantics and pragmatics of plurality are applied independently of the meanings of individual nouns.

However, other languages have morphological systems using exponents that vary with semantically motivated noun classes. Table 1 summarizes the nine classes distinguished by Harbour (2008, 2011) for Kiowa, an endangered Tanoan language spoken in Oklahoma. Bantu languages are also well-known for their large numbers of semantically motivated noun classes (see, e.g., Polomé, 1967, for Swahili). These kinds of morphological systems are open to two interpretations. On the one hand, it is possible that these noun classes are simply a feature deployed to enable linking arguments in clauses (Foley & Van Valin, 1984), and that the semantics of plurality are exactly the same for each noun class. On the other hand, as argued by Harbour (2008), number in these languages could be ‘morphosemantic’, such that different plural exponents would express related yet distinct plural semantics.

**Table 1:** Kiowa noun classes based on Harbour (2011, 2008)

Class	Semantic characteristics	Example
1	First person only	‘I’
2	Animates	‘boy’, ‘bird’
	and independently mobile inanimates	‘leg’, ‘moon’
3	Default for vegetation	‘grass’
	and implements	‘pencil’
4	Vegetation forming natural collections	‘tree’
	and implements that act collectively	‘ember’
5	Hair types	‘eyelash’
	and midsize fruit growing in clusters	‘tomato’
6	Individuable objects	‘river’
7	Non-granular mass nouns	‘water’
8	Pluralia tantum nouns,	‘trousers’
	composite nouns	‘necklace’
	and granular mass nouns	‘rice’
9	Default	‘shoe’

The first aim of this study is to show that although English does not grammaticalize semantic classes in ways similar to Bantu or Kiowa, if we look carefully at the relations between singulars and plurals in a high-dimensional semantic space using distributional semantics, we find that the changes in semantics from singular to plural nouns actually vary with the semantic class of the noun, in ways that are reminiscent of the noun classes of Kiowa and Bantu. In other words, borrowing terminology from Bresnan et al. (2001), we argue that what are hard, grammaticalized constraints in Kiowa and Swahili are soft, probabilistic constraints in English. The hard constraints in the former two languages force speakers to assign highly variegated meanings to discrete categories represented by different exponents. By contrast, English can be viewed as a system with ‘soft categorization’ that exists in the semantic system, but that is not distrectized morphologically.

The second aim of this study is to clarify whether the variegated palette of soft pluralization in English can be accounted for with a single mathematical operation in the high-dimensional space in which words’ semantic vectors are defined. To this end, we consider a model originally developed for derivation, FRACSS (Marelli & Baroni, 2015), and apply it to English plural inflection. The FRACSS model implements the idea that there is a general pluralization operation that changes singulars into their corresponding plurals. Although a FRACSS mapping in distributional space is much more complex than a straightforward proportional analogy, such a mapping stays very close to the idea that pluralization is a unified semantic operation that does not need to be informed about the specific semantic class that a noun belongs to. We contrast this application of FRACSS to inflection with an alternative model that predicts the meaning of a noun’s plural through explicit conditioning on the semantic class of the noun. We will show that the latter model has certain advantages over the FRACSS-based model.

The remainder of this study is structured as follows. We first introduce some basic concepts from distributional semantics and show how a general proportional analogy for pluralization is formalized within this framework. We then introduce the dataset that we have used for our analyses. Next, we show that plural semantics are different across semantic classes. Subsequently, we compare the FRACSS model with a class-conditioned model of pluralization. The study ends with a general discussion.

## Distributional semantics

Distributional Semantics (DS) represents words’ meanings with high-dimensional numeric vectors, which we will refer to primarily as ‘semantic vectors’ and alternatively as ‘word embeddings’ — as they are known in Natural Language Processing (NLP). Distributional semantics builds on the hypotheses that words that are similar in meaning occur in similar contexts (Firth, 1968; Harris, 1954; Rubenstein & Goodenough, 1965) and “words that occur in the same contexts tend to have similar meaning” (Pantel, 2005).

### Constructing semantic vectors

There are many different ways in which semantic vectors for words can be constructed. Early implementations made use of word-by-document contingency tables (Landauer & Dumais, 1997) or word-by-context-word contingency tables (Lund & Burgess, 1996; Shaoul & Westbury, 2010). These tables typically yield very high-dimensional vectors with thousands or tens of thousands of dimensions. By means of dimensionality reduction techniques such as singular value decomposition, the dimensionality of semantic vectors is substantially reduced. Landauer and Dumais (1997) recommended 300-dimensional vectors, as in their experience, lower-dimensional vectors performed with higher accuracy in a range of tasks such as synonymy detection.

More recent models make use of artificial neural networks that are trained to predict target words from the words in their immediate context (e.g., CBOW; Mikolov et al., 2013a) or to predict the words in the immediate context of a target word from that target word (e.g., Skip-gram; Mikolov et al., 2013a). A simple three-layer neural network for the Skip-gram model was implemented by Mikolov et al. (2013b), using stochastic gradient descent and back-propagation of error. The model was trained on 100 billion words from the Google News corpus, and the resulting word2vec semantic vectors were made available at https://code.google.com/archive/p/word2vec/.

Other methods for inferring word embeddings extend the word2vec methodology by incorporating character n-grams of words (fastText; Bojanowski et al., 2017) or by modifying the objective function being optimized (GloVe; Pennington et al., 2014). All these methods extract the semantic vectors purely from textual information. Other studies integrate visual information on top of that and create multi-modal embeddings (e.g., Shahmohammadi et al., 2021).

### Previous applications of semantic vectors

Word embeddings are employed to great advantage in several tasks within NLP, such as named entity recognition, part of speech tagging, sentiment analysis, word sense disambiguation (Wang et al., 2019), and in many areas of psychology and psycholinguistics (Günther et al., 2019a). Boleda (2020) discusses their relevance for theoretical linguistics in the areas of diachronic semantic change, polysemy, and the interface between semantics and syntax or semantics and morphology.

The traditional demarcation of morphology and semantics in linguistics is less prominent in DS models. Nevertheless, the distributional statistics used in these models have been shown to encode morphological and syntactic information besides semantic information (Westbury & Hollis, 2019). For morphologically related words, measurements from DS models, such as vector similarity, are consistent with human semantic similarity ratings and lexical decision latencies (Rastle et al., 2000, 2004; Moscoso del Prado Martín et al., 2005; Milin et al., 2009). The degree of semantic transparency in English derivation (Marelli & Baroni, 2015) and Dutch compounds (Heylen & De Hertog, 2012) has been quantified with DS-based similarity measures. Findings of Smolka et al. (2014) regarding the effect of semantic transparency on morphological priming of German complex verbs were replicated with DS similarity measures by Padó et al. (2015) (although Shafaei-Bajestan, 2017, could not fully replicate the latter study) (see also Baayen & Smolka, 2020). Shen and Baayen (2021) have reported that semantic transparency measured by DS is linked to the productivity of adjective–noun compounds in Mandarin. DS models used in investigating the paradigmatic relation between two Indonesian prefixes (Denistia et al., 2021) corroborated the findings of earlier corpus-based analyses. The discriminative lexicon model of Baayen et al. (2019) is a computational model of lexical processing, including the processing of morphologically complex words, that incorporates insights from distributional semantics for the representation of word meanings.

DS models from machine learning produce semantic vectors for both singular and plural word forms. However, in order to be useful for the study of morphology, we need to consider additional questions: What does the process of English pluralization, i.e., going from the singular to the plural semantics, mean in the high-dimensional semantic spaces in which semantic vectors are defined? How can one model the semantics of pluralization? Given a singular meaning, can we conceptualize the plural, and conversely, given the plural meaning, can we conceptualize the singular? As it is more likely that we encounter previously unseen plurals of known singulars than previously unseen singulars given known plurals, we focus specifically on the productivity of the conceptualization of plural forms and ask: How well can we estimate the semantics of previously unseen plural words?

### Proportional analogies with word embeddings

Analogical reasoning using word embeddings has been studied for different types of analogical relations, including semantic analogies, such as

$$ man:king::woman:queen, $$

derivational analogies, as in

$$ quiet:quietly::happy:happily, $$

and inflectional analogies similar to

1 $$ pen:pens::table:tables. $$

Various implementations of proportional analogies with word embeddings have been worked out (e.g., Mikolov et al., 2013c; Levy & Goldberg, 2014; Drozd et al., 2016). Performance varies extensively for the different methods and the different types of analogical relations. Rogers et al. (2017) report that, for English, analogical reasoning with embeddings is most successful for inflectional analogies across different methods. These methods are considered below in the context of plural formation.

Most of the aforementioned methods operate on three input vectors to estimate a vector for the target word in a given analogy. For instance, to implement the analogy in (1), the method proposed by Mikolov et al. (2013c) predicts a vector for *tables*, labeled $\overrightarrow{tables}_{p}$, by computing

2 $$ \overrightarrow{tables}_{p}= \overrightarrow{pens}- \overrightarrow{pen}+\overrightarrow{table}. $$

The word selected as the predicted plural is the word the vector of which is closest to the composed vector, $\overrightarrow{tables}_{p}$ in (2), in terms of cosine similarity. As a consequence, evaluation of these methods is restricted to predefined analogy test sets such as Google’s (Mikolov et al., 2013a) which provide a series of analogies similar to the examples above. Another limitation of these methods is that their prediction for the target word *tables* highly depends on the prime word pair, here *pen* and *pens*, and not on just the singular word *table* (Rogers et al., 2017). Thus, the predicted plural vector for *tables* is different when the prediction builds on another analogy such as

$$ banana:bananas::table:tables. $$

One of the two methods proposed by Drozd et al. (2016), called three cosines average (3CosAvg), on the other hand, operates on just one input vector, the vector of the base word. Given the input word *table*, the predicted plural vector by 3CosAvg is 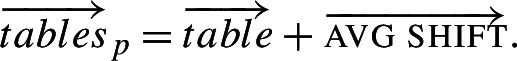


The word selected as the plural form is again exactly as for the method of Mikolov et al. (2013c), that word the vector of which is closest to the computed vector. For plural analogies, Drozd et al. (2016) define the average shift vector as 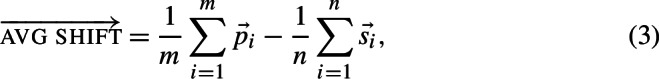
assuming there are *m* plural word-forms with vectors $\vec{p_{i}}$ and *n* singular word-forms with vectors $\vec{s_{i}}$. The average shift vector is fixed given the data, and represents the semantics of pluralization.

**Fig. 1:**
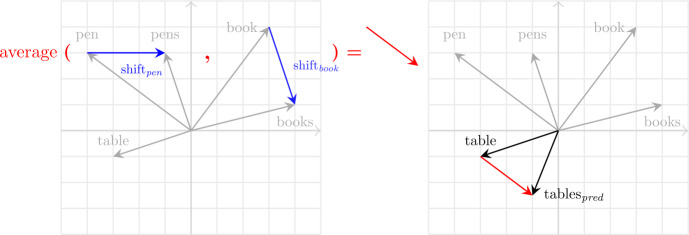
**The 3CosAvg methodology.** Left: The individual shift vector for the lexeme *pen*, depicted in blue and labeled as shift_pen_, is calculated as $\overrightarrow{\text{pens}} - \overrightarrow{\text{pen}}$. The average shift vector in red is computed over the individual shift vectors. Right: The average shift vector is used to predict a plural vector for the singular *table*

For a dataset with *m* plural and *m* singular word forms, the average shift vector, i.e., the difference vector between the average vector of plurals and the average vector of singulars, formulated in (4), is equal to the average vector of the difference vectors between plurals and singulars, formulated in (5): 
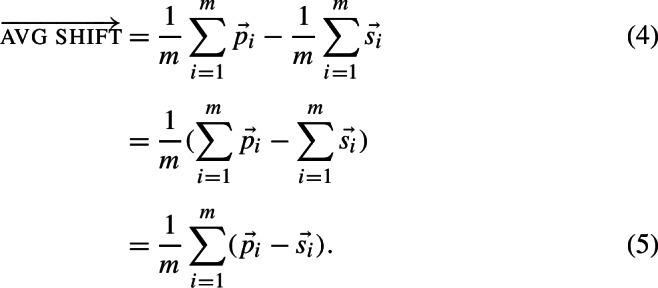
 Henceforth, we refer to the vector $\vec{p_{i}} - \vec{s_{i}}$ for word *i* as this word’s individual *shift vector*. In Fig. 1, such shift vectors are illustrated in blue for two lexemes, *pen* and *book*, within a toy 2D space. The computation of the average shift vector in this space is depicted in red on the left subplot, and the prediction using the average shift vector is visualized on the right subplot. Importantly, if plural and singular forms for different lexemes are consistently used across similar contexts, as captured by word embeddings, then the difference between individual shift vectors and the average shift vector is expected to be small.

A range of studies have adopted shift vectors to study the semantics of various lexical relations. For instance, Roller et al. (2014) and Weeds et al. (2014) used shift vectors for hypernymy detection. Bonami and Paperno (2018) used shift vectors to model inflectional and derivational contrasts in French, and Mickus et al. (2019) made use of shift vectors for tracing contrasts in the grammatical gender of nouns and adjectives.

In the following section, we introduce the corpus that we used in the present study to examine whether an average shift vector provides an optimal approximation of the semantics of pluralization in English within the framework of distributional semantics.

## Data

The corpus data used in this study is taken from the NewsScape English Corpus (Uhrig, 2018, 2022), which is suitable for this study for multiple reasons. First, the corpus is quite large. It consists of 269 million tokens from the subtitles of more than 35,000 hours of recordings of US-American TV news collected in the UCLA Library Broadcast NewsScape (Steen et al., 2018). Secondly, it is relatively varied. The shows in the corpus cover a range of different levels of formality and different registers, ranging from relatively casual daytime chat shows to evening news, which follow a very rigid format. Similarly, the speakers come from various regional backgrounds, ethnicities and are of various ages and genders, although there is likely to be a bias towards older white men, which any representative sample of American TV will show. In addition to being quite large, it provides transcripts in the form of subtitles, which are aligned with the audio signal so that we can extract auditory features, and it sports automatically generated phonetic transcriptions, which we use for our triphone approach. The processing steps are briefly summarized below. Third, for all words, we have the audio files, which make it possible to study mappings between words’ acoustic signals and their semantics (see, e.g., Shafaei-Bajestan et al., 2021, 2023).

After capture, the recordings undergo compression, during which the audio channel is recoded into a 96 kbit/sec AAC stream with the Fraunhofer FDK library. For this project, the subtitles collected in the NewsScape text files were processed in an NLP pipeline.

In the first step of this pipeline, sentence splitting was carried out with a purpose-built splitter that takes into account the fact that captions are transmitted in upper case. The resulting sentences were processed with Stanford CoreNLP (Manning et al., 2014) version 3.7.0, i.e. with PTB3 tokenization. The caseless model included in CoreNLP1 was used to tag every word with a Penn Treebank part-of-speech tag.^1^ Then CoreNLP’s TrueCase annotator was deployed, which overwrites the original text for further processing (preserving the original on a separate level). Dependency Parsing, Named-Entity Recognition, and any further processing steps are then based on the case-restored text to ensure consistent results from tools that do not offer caseless models.

After the NLP pipeline, the data was run through a modified version of the forced alignment system Gentle (Ochshorn & Hawkins, 2015), which basically runs an automatic speech recognition process with a language model created from the subtitles and then attempts to match the recognized words with the words in the subtitles. The quality of the forced alignment results crucially depends on the accuracy of the transcript it is fed. However, TV subtitles are not exact transcripts. Not only do they often ignore disfluencies such as false starts, but they also omit words and sometimes even change them. The commercials included in the recordings do not systematically come with subtitles either. Thus, on average, Gentle only aligns between 90 and 95 percent of the words in the subtitles successfully, and of these, 92.5% in a manual evaluation were deemed to be aligned correctly by a human annotator listening to them (Uhrig, 2022). We have to bear in mind, though that the cutoff points may not have been exact on these words. To increase the quality of the dataset used in the present study, only files where Gentle reported at least 97% of successfully aligned words were used.

Words’ meanings are represented with the pre-trained word2vec semantic vectors distributed by Mikolov et al. (2013a), which is widely used within NLP and theoretical linguistics. The nearest neighbors of a target word in this semantic space are often semantically similar (e.g., *good* and *great*) or related (*good* and *bad*) words. The top 10 closest neighbors to *Germany*, for instance, are *German*, *Europe*, *European*, *Sweden*, *Switzerland*, *Austria*, *France*, *Spain*, *Poland*, and *Russia*. Wang et al. (2019) show that similarities computed between pairs of word2vec vectors are highly correlated (*r*(2998)=0.72) with similarity ratings between word pairs obtained from human subjects in the MEN data set (Bruni et al., 2014), and that word2vec vectors are best performing on syntactic word analogy tasks juxtaposed with five other semantic spaces. Westbury and Hollis (2019) argue that Mikolov et al. (2013a)’s approach for training of the word2vec vectors is closely related to the cognitively plausible learning rule of Rescorla and Wagner (1972).

We compiled a *noun pluralization* dataset with 14,699 singular-plural noun pairs from the NewsScape English Corpus with a word2vec vector. Proper names, plurals ending with anything other than an *-s*, plural-singular pairs with the same word form, and named entities were excluded from the dataset. There are 29,303 unique singular and plural nouns in this dataset.

A second set brought together a distinct set of 6569 orthographic word types also extracted from the NewsScape English Corpus. These words, forming our *vocabulary* dataset, were chosen without constraints on their morphological and syntactic categories. For instance, different conjugated forms of the verb “to do” are included in the vocabulary dataset. None of these words in this second dataset occur in the noun pluralization dataset. Each word in the vocabulary dataset has a corresponding word2vec vector. This dataset is used below to expand the range of possible predictions during the evaluation of model performance. Taken together, the two datasets comprise 35,872 unique words.

## Analysis of plural shift vectors

Recall that the average shift vector has been proposed as a viable representation of plurality. However, how well does an average shift vector approximate the shifts between individual singulars and their plurals? To address this question, we first investigated what the individual shift vectors look like and whether the average shift vector is representative of the individual shift vectors.

### The average shift vector

For each noun pair in the pluralization dataset represented by word2vec semantic vectors, we calculated its individual shift vector by subtracting the singular vector from the plural vector. The average shift vector was obtained by averaging the individual shift vectors according to Equation (5). Subsequently, we computed the length (or magnitude), the direction, and the neighborhood structure of the average and the individual shift vectors.

**Fig. 2:**
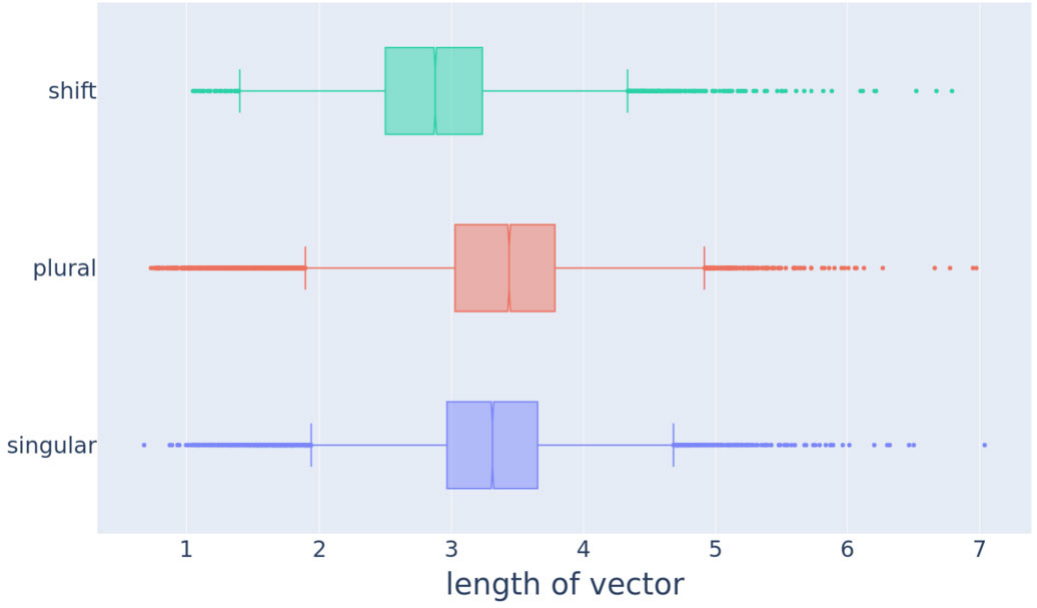
Boxplots for the length of 14,699 word2vec’s singular, plural, and individual shift vectors

We gauged the length of vectors with the $\ell _{2}$ norm, i.e., the Euclidean distance of a vector from the origin. Figure 2 shows box and whiskers plots for the length of singular, plural, and individual shift vectors. Vector lengths differed in the mean for singular, plural, and shift vectors (Friedman test, $\tilde{\chi}^{2}(2) = 7201$, *p*≪0.0001). Pairwise Wilcoxon signed-rank test between groups with Bonferroni correction revealed significant differences in length for all pairwise comparisons (all *p*≪0.0001). Plural vectors are, on average, longer than singular vectors (the difference between the medians ΔMD is 0.13).

The interpretation of the length of word vectors in word2vec models has been a subject of debate among researchers. Some argue that vector length primarily corresponds to word frequency (e.g., Jurafsky & Martin, 2021, Chap. 6), while others propose that it additionally indicates similarity in contextual usage. Schakel and Wilson (2015), Wilson and Schakel (2015) provide experimental support for the latter viewpoint, demonstrating that words consistently appearing in similar contexts are represented by longer vectors, distinguishing them from words with the same frequency but different contexts. This finding justifies the use of word vector length as a measure of word significance^2^ or a measure of the absence of co-occurrence noise, as longer vectors are indicative of a distinctive context.

We can rule out the possibility that longer plural vectors result from higher frequencies of plural words. In fact, it is commonly observed that plural forms are often less frequent compared to their singular counterparts in numerous languages (see, e.g., Baayen et al., 1997, for Dutch and the processing consequences of frequency and plurality). To corroborate this, we conducted an analysis using data from the Corpus of Contemporary American English (COCA; Davies, 2010). We used the academic, fiction, magazine, newspaper, and spoken sections of COCA 1990-2012 with nearly 450 million words to obtain frequency counts that closely reflect actual usage in a broader range of registers than sampled by the NewsScape corpus.

**Fig. 3:**
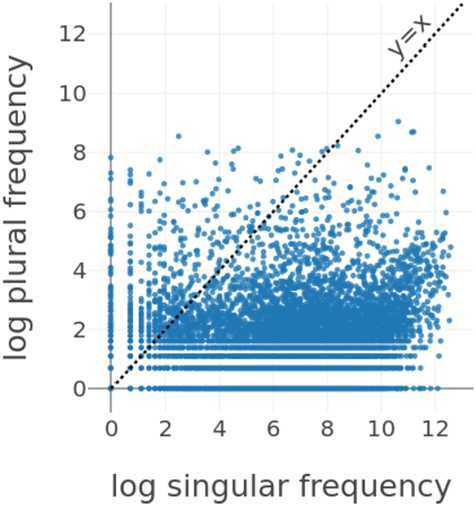
Scatter plot for the logarithm of the plural frequency as a function of the logarithm of the singular frequency for 7891 English lexemes from our pluralization data set that occur in COCA. The dashed black line shows the identity line *y* = *x*

The counts from COCA indicate that plurals tend to have lower frequencies than their corresponding singulars. As illustrated in Fig. 3, which represents the log COCA frequency of the plural form against the log COCA frequency of the singular form for 7891 English lexemes, the majority of the data points fall below that identity line. That is, the majority of the lexemes have a larger frequency in the singular form than in the plural form. All these lexemes were selected from the pluralization dataset with the condition that both the singular and plural forms were present in COCA.

Taken together, the finding that plural vectors are longer than singular vectors indicates that English plurals occur in less diverse potential contexts. The constraint on the number of possible interpretations increases the discriminability of the plural meaning and fits well with the proposal that plurals are semantically marked (de Swart & Farkas, 2010).

Shift vectors are, on average, smaller than the singular (ΔMD = 0.43) and the plural vectors (ΔMD = 0.56), which is only to be expected given that the shift vectors are, by definition, difference vectors. Although the average length of the shift vectors is smaller than the average lengths of singular or plural vectors, shift vectors turn out to nevertheless be surprisingly long. Their range (1.1 − 6.8, MD = 2.88) is nearly as wide as the ranges of the singular vectors and the plural vectors.

**Fig. 4:**
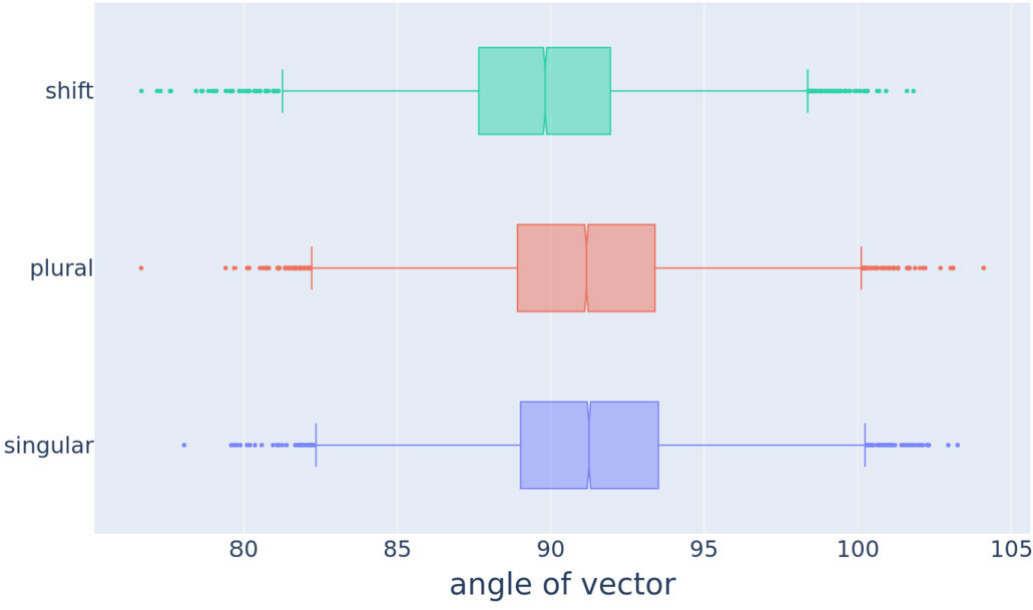
Box plots for the angle of 14,699 word2vec’s singular, plural, and individual shift vectors

We quantified the angles of vectors in word2vec’s 300-dimensional vector space with respect to the standard unit vector $\vec{e}_{300}$ in degrees, using (6). This 300-dimensional unit vector has a one as the last element and zeros elsewhere. Boxplots for angle are presented in Fig. 4. The range of angles for shift vectors is even more similar to the ranges of angles of the singular and plural vectors compared to vector lengths.

6 $$\begin{aligned} \theta (\vec{v}) &= \frac{180}{\pi} (\arccos { \frac{\vec{v} \cdot \vec{e}_{300}}{ \left \lVert \vec{v}\right \rVert _{2} \left \lVert \vec{e}_{300}\right \rVert _{2} }) } \\ &= \frac{180}{\pi} (\arccos { \frac {v_{300}}{{\sqrt {\sum \limits _{i=1}^{300}{v_{i}^{2}}}}}}). \end{aligned}$$

**Fig. 5:**
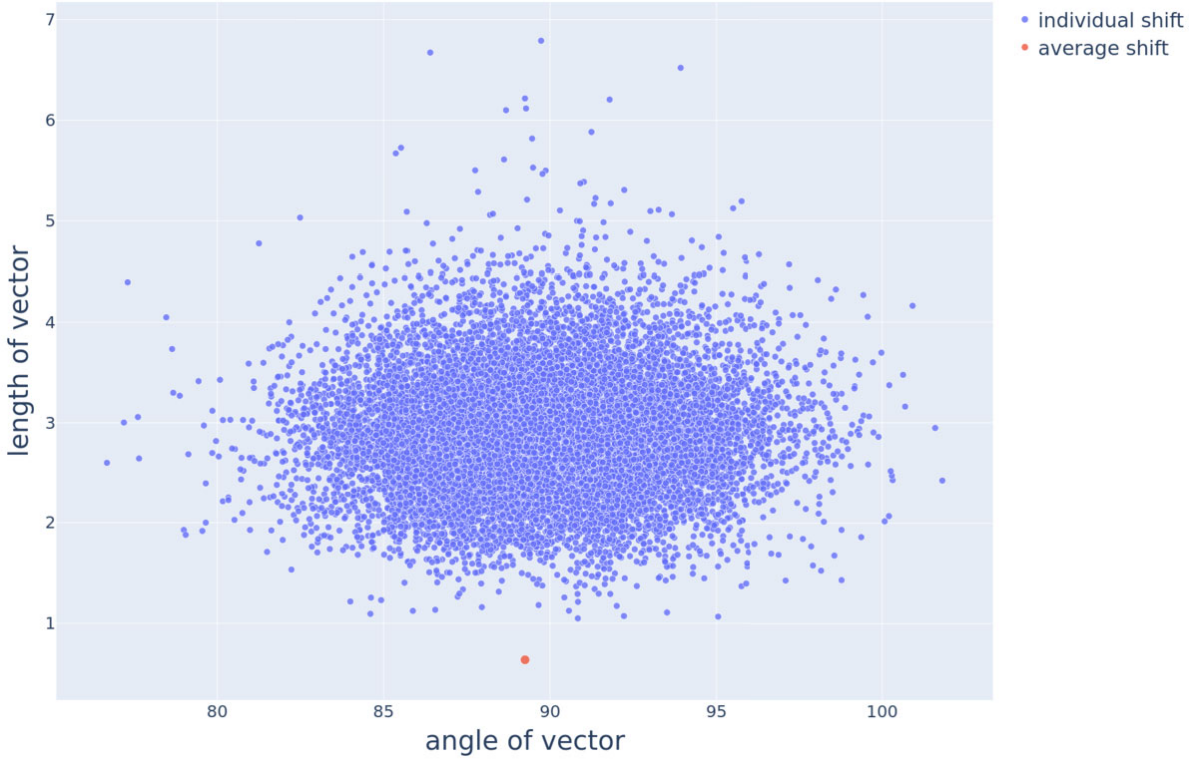
Scatter plot visualizing the relationship between the length, on the y-axis, and angle, on the x-axis, for the individual shift vectors. The isolated red dot below the cloud of all other data points at (89.25, 0.64) belongs to the average shift vector

Figure 5 plots the length of shift vectors against their angle. Considerable variability is visible in the length and the angle of individual shift vectors. The average of a set of vectors radiating from the origin that point in various directions and have various lengths will inevitably end up close to the origin of that vector space. The average shift vector, in red, at (89.25, 0.64), is smaller than all of the individual shift vectors and has an $\ell _{2}$ norm of only 0.64. When such a small vector is added to the singular, it is hardly distinguishable from the singular vector, and at a large distance from the actual corresponding plural vector.

### Clusters of shift vectors

**Fig. 6:**
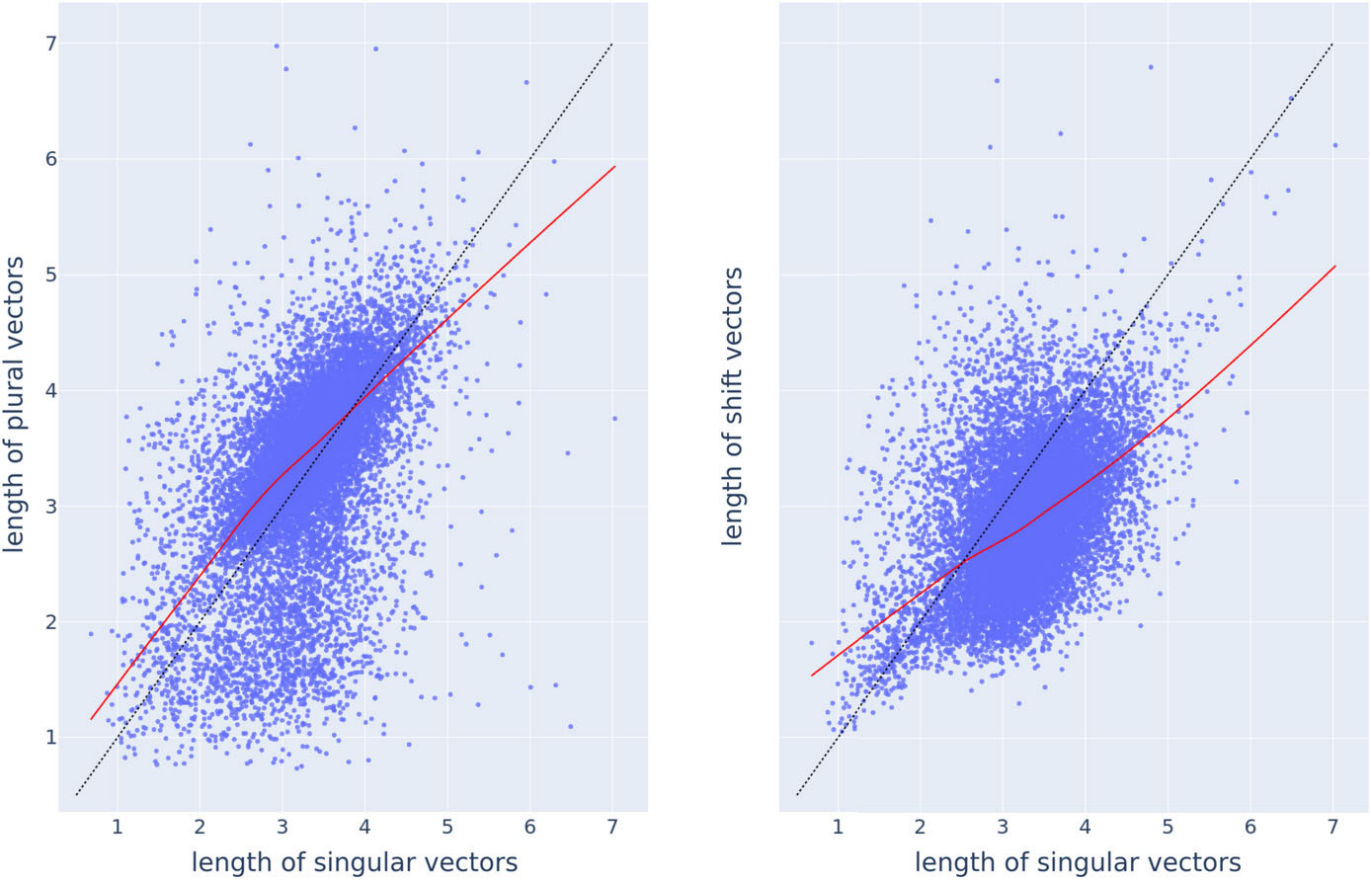
Scatterplots for the length of plural vectors (vertical axis in the left panel) and length of shift vectors (vertical axis in the right panel) against the length of singular vectors (horizontal axis in both plots), with LOcally WEighted Scatterplot Smoothing (LOWESS) trend lines depicted as solid red lines. The dashed black lines represent the identity line *y* = *x*

Upon closer inspection, it turns out that, rather than being random, the set of individual shift vectors exhibits structure. The length of plural vectors increases with the length of their singular vectors, and likewise, the length of shift vectors increases with the length of the singular vectors, as illustrated in Fig. 6. From this, we can draw the conclusion that the semantics of shift vectors is changing in close association with the semantics of the singular and plural words.

Given that singular words that have similar semantics have closer vectors, and singular words with less similar meanings have more diverging vectors, we now consider the question of whether the shift vectors themselves show structuring that goes beyond the structure provided at the level of individual lexemes. To address this question, we made use of the t-SNE algorithm for visualizing high-dimensional data (van der Maaten & Hinton, 2008) as implemented in the scikit-learn Python library (Pedregosa et al., 2011), version 1.0.1, to plot the 300-dimensional shift vectors in a two-dimensional plane.^3^ This visualization technique is known to have a very high chance of recovering the clustering structure present in the input space in the reduced output space (Linderman & Steinerberger, 2019; Arora et al., 2018).

**Fig. 7:**
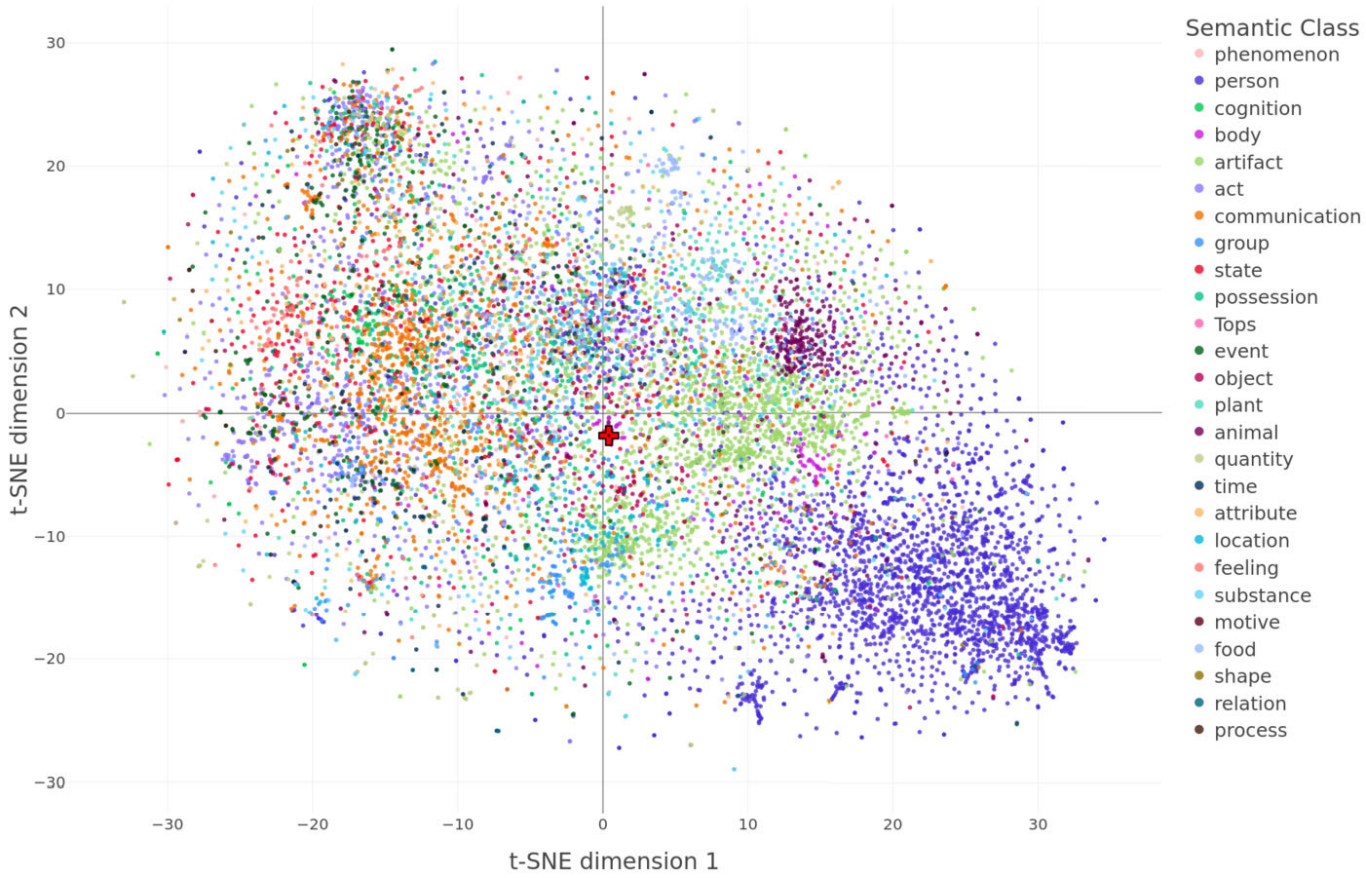
**The pluralization palette.** A projection of shift vectors onto a two-dimensional plane using t-SNE reveals semantic clustering. Colors correspond to WordNet supersenses. The average shift vector marked with a red cross, which is located very close to the origin at (0.4, −1.8), is blind to this rich structure. The interactive plot for this figure is available in the supplementary materials

Figure 7 presents the scatter of data points in this plane, colored with the label of the first synset in WordNet (Fellbaum, 1998; Miller, 1995) for the singular word form. We recommend exploring the interactive version of this plot, which is accessible in the supplementary materials. From the 14,699 pairs in our pluralization dataset, 11,749 pairs are found in WordNet and used in the remainder of this study. The labels, indicated in the figure’s legend, often referred to as WordNet supersenses, include 26 broad semantic categories for nouns (Ciaramita & Johnson, 2003). Interestingly, the individual shift vectors form clusters that are reasonably well approximated by the supersenses. Some supersenses show discernible clusters, such as *person* towards the bottom right corner of the plane in navy blue and *animal* towards the top right corner in plum purple. This indicates that pluralization is similar for nouns denoting animals and is different for nouns denoting persons. Importantly, the average shift vector (highlighted by a red cross) is located near the origin of this space at (0.4, −1.8), incapable of capturing the underlying structure.

Interpretation of the t-SNE dimensions is not very straightforward. Preliminary investigation suggests that the first dimension is, to a very large extent differentiating between concrete and abstract words (see supplementary materials for details). The second dimension is less interpretable and rather similar to the first dimension.

**Fig. 8:**
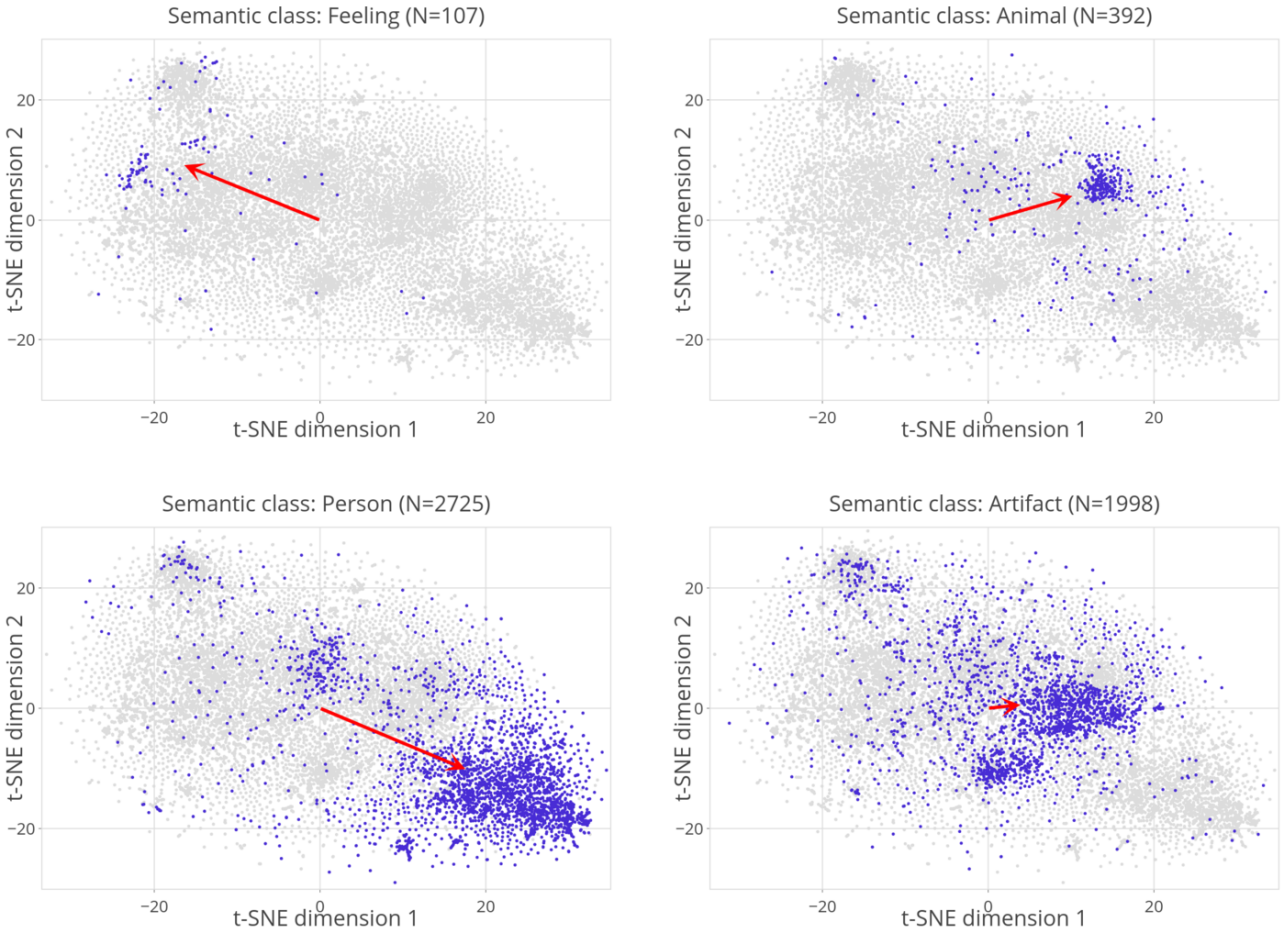
Zoomed-in scatter plots of the same data presented in Fig. 7, focused on four selected supersenses

While Fig. 7 provides a global picture, Fig. 8 offers enhanced clarity by focusing separately on selected classes. Each subplot represents data points belonging to a particular semantic category by using blue color, distinguishing them from the rest of the data points, which are depicted in gray. The semantic class and the number of blue dots are given in the title of the respective subplot. The red arrows will be discussed later. While the plots in the upper part of the figure reveal noticeable clusters for the categories of *feeling* and *animal*, there is still significant variability observed within these clusters. The lower part of the figure exhibits a considerably higher variability for the semantic classes *person* and *artifact*, revealing discernible subclusters.

The blurriness of the clusters can be primarily attributed to two underlying issues. The first issue is that nouns can have multiple senses; however, instead of having sense-specific embeddings, we currently employ word2vec vectors that provide a single embedding for all senses. Therefore, we chose one sense per word to categorize words semantically. We selected the first sense listed in WordNet, which, according to Jurafsky and Martin (2021), is the most frequent sense and hence a strong baseline. Nevertheless, inaccuracies are inevitable. For instance, despite the proximity of *strawberry* and *blueberry* in the word2vec semantic space, they are assigned to distinct semantic classes in our analysis; the former is categorized as food while the latter as a plant. This is because, according to WordNet, the primary sense attributed to *strawberry* is fruit, whereas its secondary sense is plant. Conversely, *blueberry* is classified as a plant in its primary sense, while its secondary sense is associated with being a fruit. Similar issues arise with numerous other words, including *mum*, *Chihuahua*, and *donkey*, where their primary sense is registered as the chrysanthemum flower, the Mexican state, and the symbol of the Democratic party, respectively, instead of denoting the parent, the dog, and the hoofed mammal sense, as intuitively expected.

The second issue is that the supersenses are often too broad and too over-populated to form semantically coherent groups. For instance, the supersense *artifact* brings together musical instruments, vehicles, clothes, guns, and buildings, among others. Similarly, the supersense *person* covers 2725 lexemes in our data, including relatives, job titles, insults, supernatural creatures, and more. As shown in Fig. 8, these supersenses are found in multiple distinct regions in the t-SNE plane. The fuzziness of the 26 supersenses is clearly demonstrated by Linear Discriminant Analysis (LDA) given the task of assigning shift vectors to supersenses. From an evaluation of the LDA on all of the data points (*N* = 11,749), accuracy and weighted average F-score were both 58.4%. To put the multiclass classification performance of the LDA into perspective, the weighted average F-score by the LDA is seven times greater than the weighted average F-score of a baseline classifier that always predicts the most frequent superset. The LDA’s performance indicates that, on the one hand, there is structure, and the structure is captured by both a supervised algorithm, i.e., LDA, and an unsupervised algorithm, i.e., t-SNE. On the other hand, it indicates that there is also considerable uncertainty about superset membership.

To address the first problem, one would have to make use of techniques for word-sense disambiguation. Word sense disambiguation has a very long history in computational linguistics, and many supervised and unsupervised algorithms are designed for this task. One might combine WordNet and FrameNet (Baker et al., 1998) annotations as proposed by Baker and Fellbaum (2009), train a supervised model (e.g., Zhong & Ng, 2010), or search for words’ nearest neighbors in a contextual word embeddings space (Loureiro & Jorge, 2019). Given a high-accuracy word sense disambiguation pipeline, one could then apply word sense disambiguation before calculating embeddings using word2vec. Such a program, if at all feasible, is outside the scope of the present study.

The second problem is more straightforward to address. Instead of using the 26 supersenses shown in Fig. 7 or dismissing WordNet altogether, we can zoom in on smaller, more semantically homogeneous sense sets. For instance, by moving to semantic classes one taxonomic level below the supersense *person*, we obtain more coherent subsets such as *relative*, *scientist*, and *lover*. For our pluralization dataset, we constructed a total of 411 classes by moving zero steps or one step down from the supersenses, the goal being to avoid both very large and very small class sizes. The resulting mean class size was 28.6, the range of class sizes was 5 to 481.

**Fig. 9:**
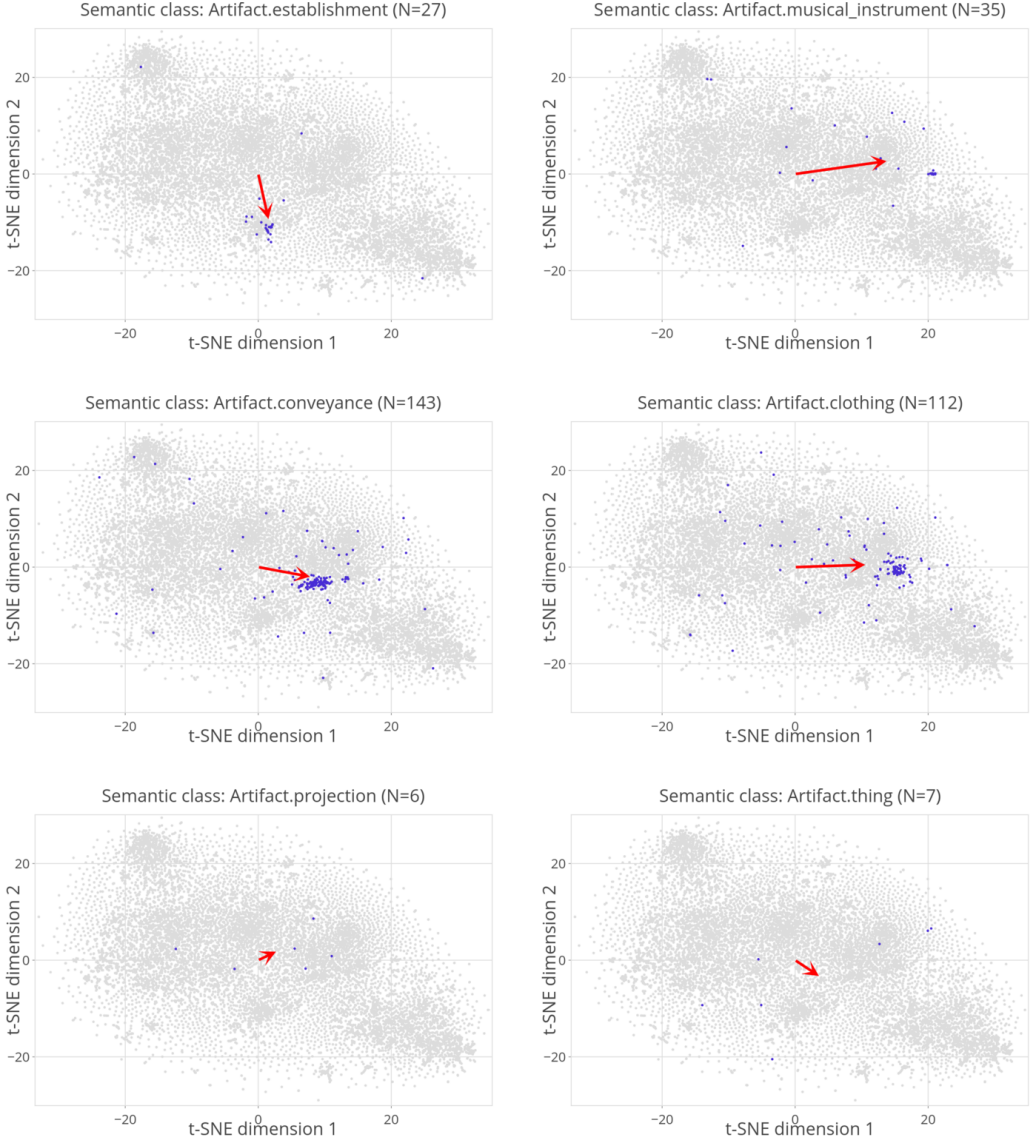
2D visualization of shift vectors using t-SNE for 6 of the 411 semantic classes obtained from WordNet. The new set of classes improves the coherence of the clusters for most categories. Nevertheless, some categories are still noisy

These new semantic classes are generally more semantically cohesive. Figure 9 presents 6 of the 75 classes that replaced the supersense *artifact* in the new set of classes. The top row shows two classes in which one cohesive cluster is visible, and the majority of points belong to that cluster. Nevertheless, the new classes are not noise-free either. The middle row illustrates classes where a major cluster is discernible while noise is substantial. The bottom row depicts two classes that are conceptually still too broad (e.g., *thing*) and, therefore, critically characterized by noise. See supplementary materials for a complete list of the words and their assigned categories. Overall, the performance of LDA increased despite the substantial increase in the number of classes. Accuracy and weighted average F-score are both 61% from an evaluation of an LDA that predicts 411 classes given the shift vectors. In comparison, the weighted average F-score by this model is 189 times greater than the weighted average F-score of a baseline classifier that always predicts the most frequent class.

Although the simplicity of having a single abstract semantic operation for plurality, formalized with the average shift vector, is appealing, such a simple average shift vector fails to do justice to the intricate distributional structure that characterizes nominal pluralization in English. The semantics of English pluralization is substantially more subtle and varies systematically with semantic categories (supersense) of nouns. Using the terminology of Bresnan et al. (2001), English pluralization is governed by a soft, probabilistic semantic constraint that is similar to the hard constraints found in the grammar of Kiowa and Bantu languages.

### Formalization: the CosClassAvg model

This new set of 411 classes, or a similarly cohesive set of classes of semantically related words, makes it possible to formulate a new model for plural semantics.

To set up this model, we first calculated the average shift vector for each of the 411 classes. The red arrows drawn in Fig. 8 and Fig. 9 depict average shift vectors for the respective semantic class. Despite significant variability within many classes, the average shift vectors consistently align with the predominant direction of the data points, indicating their collective tendency (see the first four subplots in Fig. 9). Occasionally, as observed in cases such as the last two subplots in Fig. 9, the data points are scattered across the semantic space, and the average vector settles midway in an uninterpretable direction.

The mean length of the class-specific average shift vectors is 1.2, and its standard deviation is 0.3. Compared to the distribution of shift vectors shown in Fig. 2, both mean and standard deviation are substantially reduced. The same holds for their angles (*M* = 89.1, SD = 2.6). This clarifies that by-class shift vectors are more similar to each other than is the case for the shift vectors in the undifferentiated set of all nouns.

We can now introduce our Cosine Class Average (CosClassAvg) theory for noun plurals. Given an input word and its semantic class, the plural vector predicted by CosClassAvg is obtained by taking the singular vector and adding to it the average shift vector for that class. Thus, the vector for *bananas* is predicted using 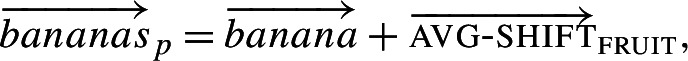
while the vector for *cars* is predicted based on 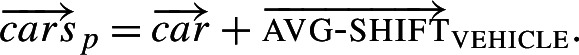


How well does CosClassAvg perform? To address this question, we first investigated whether predicted plurals are better differentiated from their singular counterparts. As our baseline for comparisons, we used the Only-b method introduced in Linzen (2016), where b represents the vector for the base word. This method simply returns the input singular vector, without adding anything to it, as the predicted plural vector. As a consequence, this method will always predict the nearest neighbor in terms of cosine similarity, i.e., the word — possibly inflected — that is most similar to the base word in the vocabulary.

We calculated the predicted plural vectors for all singular words in our pluralization dataset (*N* = 11,749) using 3CosAvg, CosClassAvg, and the baseline method. Many implementations of proportional analogies with word embeddings exclude the input words, such as the singular word from the vocabulary, as a potential predicted word. However, in an “honest” practice, as Rogers et al. (2017) put it, we do not exclude any words from the vocabulary. Therefore, we compared predicted vectors with a broader set of words covering all 30,497 word-form types in our pluralization and our vocabulary datasets.

**Fig. 10:**
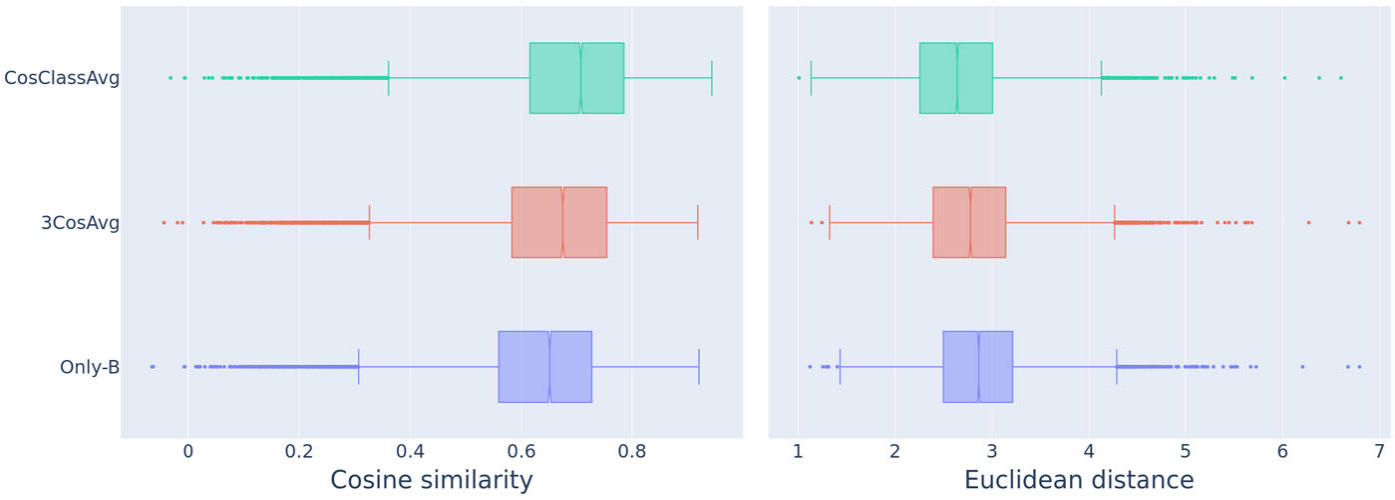
Comparison between the predicted plural vectors and the corpus-extracted plural vectors using cosine similarity (left panel) and Euclidean distance (right) for the Only-B, the 3CosAvg, and the CosClassAvg method

The boxplots in Fig. 10 summarize the distributions of cosine similarities (left) and Euclidean distances (right) for the baseline model (Only-B), the 3CosAvg model, and the new CosClassAvg model, of the predicted vectors and the corresponding plural vectors provided by word2vec. The lowest boxplots in blue produced by the baseline method indicate that the singular and the plural vectors in word2vec are already astonishingly similar. Both 3CosAvg and CosClassAvg improve on the baseline and generate more similar and less distant vectors to the actual plural vector, with CosClassAvg in the lead.

**Fig. 11:**
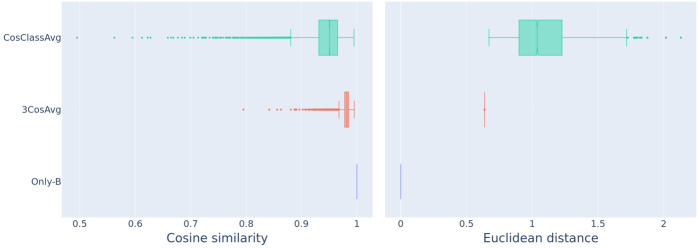
Comparison between the predicted plural vectors and the singular vectors using cosine similarity (left panel) and Euclidean distance (right) for the Only-B, the 3CosAvg, and the CosClassAvg method

For predicted vectors to well approximate the true plural vectors, they should be less close to their corresponding singular vectors. Figure 11 visualizes cosine similarity to singular vectors and Euclidean distance from singular vectors of the predicted plural vectors. Similarity increases from Only-B to 3CosAvg, and further from 3CosAvg to CosClassAvg. Conversely, Euclidean distance decreases from Only-B to 3CosAvg to CosClassAvg.

When we use the stringent criterion that any word, including the singular, can be a neighbor of the predicted plural, then the performance of both 3CosAvg and CosClassAvg is disappointing. 3CosAvg always selects the singular as the closest neighbor, and CosClassAvg only correctly selects 42 plurals (0.4%). Although CosClassAvg yields predicted vectors that are further away from their singulars and closer to their plurals, compared to 3CosAvg, predicted plural vectors remain very close to their singular vectors. Table 2 lists the percentages of lexemes for which the targeted plural vector is among the top-n neighbors. Of the three methods, CosClassAvg clearly outperforms the other two, with percentages ranging from 79% to 95%. In other words, if we relax our criterion and filter out singular vectors as candidates, the accuracy of CosClassAvg is at 79%.

**Table 2:** Percentage of the lexemes (*N* = 11,749) for which the plural vector is the first (Top 1) or among the 2 (Top 2), 3 (Top 3), 10 (Top 10), and 20 nearest neighbors (Top 20) of the predicted plural vector

Method	Top 1	Top 2	Top 3	Top 10	Top 20
Only-B	0	61	74	88	92
3CosAvg	0	70	80	91	93
CosClassAvg	0.4	79	86	93	95

### Discussion

According to the 3CosAvg method proposed by Drozd et al. (2016), pluralization can be formalized as a function adding an *average shift vector* to the singular vector: 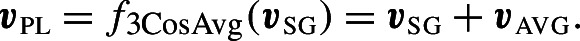
We have shown that this formalization of plurality is too simple, as shift vectors form semantically motivated clusters. The CosClassAvg model takes these classes into consideration using a conditional plural shift vector: 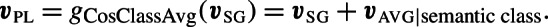


Pluralization with CosClassAvg requires two pieces of information to make a prediction, namely, information on the semantic clusters (and their centroids) and information on the semantic class membership of a given singular noun. The current study shows that, given this information, more precise predictions for plural vectors are obtained.

These predictions, however, are noisy for several reasons. First, within a given category, such as the category of fruits, *apples* and *oranges* are more similar in how they occur together than *apples* and *bananas*.^4^ This leads to within-class variation that is outside the scope of the CosClassAvg model. The vectors predicted by CosClassAvg will always lack precision for individual words. We, therefore, update the plural semantic function *g* with a lexeme-specific error term as follows: 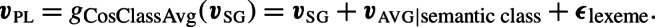
The error vector $\boldsymbol{\epsilon}_{\text{lexeme}}$ represents the lexeme-specific semantics that cannot be captured by the semantic commonalities of the lexeme’s semantic class. In usage-based grammar and corpus linguistics, individual words, including inflected words, have been argued to have their own highly specific usage profiles (see, e.g., Sinclair, 1991). ‘Error’ components such as $\boldsymbol{\epsilon}_{\text{lexeme}}$ formalize this important insight.

Second, as illustrated by Fig. 9, there is considerable variability within and across clusters of shift vectors. As explained above, this is due, at least in part, to a lack of precision in the way we assigned words to semantic classes: for a given word, we took its most highly-ranked class assignment. Furthermore, the semantic classes provided by WordNet are reasonable but not perfect, and the word2vec embeddings that we made use of, being measurements, are also not free of measurement error. We, therefore, add a second error term that represents the noise that is inherent to the data collection process: 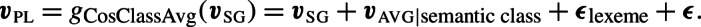
For a similar model applied to Finnish nouns, where shift vectors have recently been shown to be conditional on case, and which also proposes these two error terms, refer to Nikolaev et al. (2023).

## Alternative approaches to pluralization

The CosClassAvg model formalizes the observation that plural shift vectors change with semantic class. This model thus departs from the idea that pluralization is symbolic, in the sense of a semantic function that has the same effect, irrespective of the lexeme that is provided to it as an argument. Hence, its argument can be conceptualized as a symbol (see also the quote from Lyons (1968) in the introduction, in which the variable *x* is the symbol that the analogy has to evaluate).

### Pluralization with FRACSS

However, there is another way in which the semantics of noun pluralization can be approached, namely, by means of a single semantic operation (other than adding the average shift vector as in 3CosAvg) in the form of a mapping from singulars to plurals. This kind of mapping was proposed for derivational morphology by Marelli and Baroni (2015), building on previous research on compositional semantics (Mitchell & Lapata, 2008; Baroni & Zamparelli, 2010; Lazaridou et al., 2013). Their FRACSS model has been applied to German complex verbs (Günther et al., 2019b), and an extended version has been used to study compounding in English and German (Günther & Marelli, 2016, 2019; Marelli et al., 2017; Günther et al., 2020). The FRACSS model also lends itself for application to the semantics of plural inflection. Formally, a mapping matrix ***B*** is used to transform a singular vector into its plural counterpart. 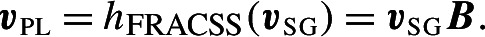


#### Technical details

To see how ***B*** is calculated, let ***X*** denote a matrix with as row vectors the word embeddings of singulars, and let ***Y*** denote a matrix with the same number of row and column vectors representing the meanings of the corresponding plurals:

$$ {\displaystyle \boldsymbol{X}= { \begin{pmatrix} x_{1,1} & x_{1,2} & \cdots & x_{1,n} \\ x_{2,1} & x_{2,2} & \cdots & x_{2,n} \\ \vdots &\vdots &\ddots & \vdots \\ x_{t,1}&x_{t,2}&\cdots & x_{t,n} \end{pmatrix} }, \quad \boldsymbol{Y}= { \begin{pmatrix} y_{1,1} & y_{1,2} & \cdots & y_{1,n} \\ y_{2,1} & y_{2,2} & \cdots & y_{2,n} \\ \vdots & \vdots & \ddots & \vdots \\ y_{t,1} & y_{t,2} & \cdots & y_{t,n} \end{pmatrix} }. } $$

The mapping ***B*** is a *n* × *n* dimensional matrix that satisfies

$$ \boldsymbol{X} \boldsymbol{B} = \boldsymbol{Y}. $$

We solve for ***B*** using the normal equations of the linear regression model as follows:

$$ \boldsymbol{B} = (\boldsymbol{X}^{T} \boldsymbol{X})^{-1} \boldsymbol{X}^{T} \boldsymbol{Y}, $$

where $\boldsymbol{X}^{T}$ is the transpose of ***X***, and $(.)^{-1}$ denotes a matrix inverse operation. Given ***B*** and the vector of a singular, the predicted plural vector is given by

$$ {\displaystyle { \begin{bmatrix} x_{1} & x_{2} & \cdots & x_{n} \end{bmatrix} } { \begin{pmatrix} b_{1,1} & b_{1,2} & \cdots &b_{1,n} \\ b_{2,1} & b_{2,2} & \cdots &b_{2,n} \\ \vdots &\vdots &\ddots &\vdots \\ b_{n,1} &b_{n,2}&\cdots &b_{n,n} \end{pmatrix} } = { \begin{bmatrix} \hat{y}_{1} & \hat{y}_{2} & \cdots & \hat{y}_{n} \end{bmatrix} }, } $$

which, according to the definition of matrix multiplication, implies that

$$\hat{y}_{j} = \sum _{i=1}^{n} b_{i,j} \cdot x_{i}, \qquad {\text{for } 1\leq j \leq n }. $$

In other words, the *j*-th element of the semantic vector of a given plural is a weighted sum of the values of its singular vector.

#### Evaluation on the pluralization dataset

We estimated the mapping matrix ***B*** for 90% of the singular-plural pairs in our pluralization dataset (10,574 pairs) using 300-dimensional word2vec vectors. The remaining 1175 word pairs were set aside as held-out testing data. The resulting 300 × 300 ***B*** matrix implements the change in the meaning of singular words that goes hand in hand with the affixation of the plural *-s*. With ***B*** in hand, we can calculate predicted plural vectors for both the training data and the test data. For evaluation, a brute-force similarity search finds the nearest gold standard plural vector, among all vectors in ***Y***, to the predicted plural vector in terms of Pearson’s correlation coefficient. The predicted plural form is the word with the highest correlated vector. The model correctly predicts plural forms for 88% of training items and for 76% of test items. Clearly, the mapping appears robust as a memory for seen items, and it is also productive for unseen items.

**Fig. 12:**
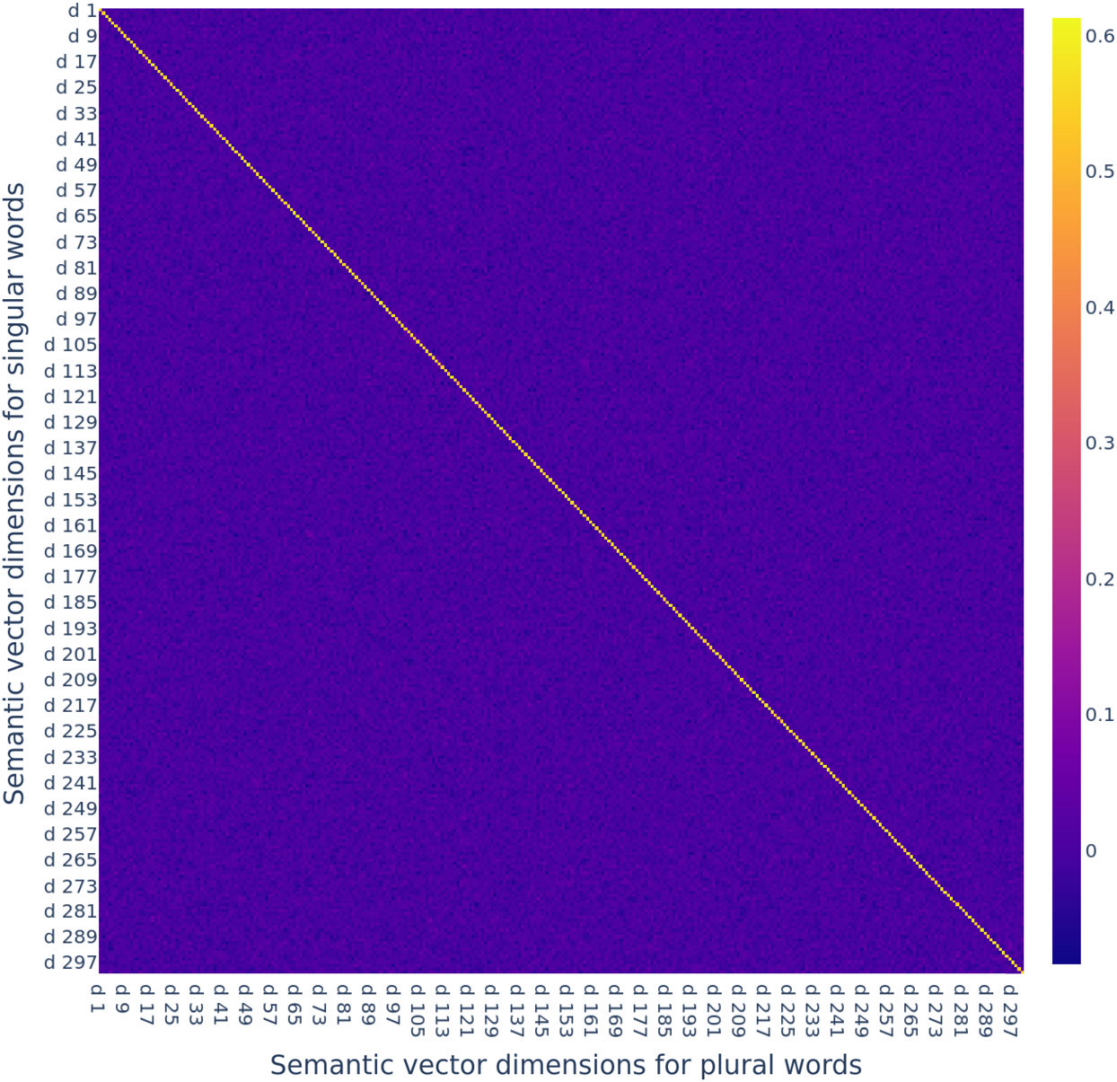
FRACSS matrix for English plural suffix -s

To better understand the performance of the FRACSS model, recall that word2vec’s singular and plural vectors are very similar. That is to say, any model for finding a mapping between the singular and the plural space is a priori in an advantageous position since the relationship between the two spaces is already a given property of the semantic space constructed by word2vec. In other words, the mapping matrix ***B*** must be somewhat similar to an identity matrix (i.e., a matrix with ones on the diagonal and zeroes elsewhere). The cool-to-warm heat map in Fig. 12, which visualizes the FRACSS matrix, shows that this is indeed the case. Input vector dimensions are on the vertical axis, indexed by *i* from 1 to 300, and output vector dimensions, indexed by *j* from 1 to 300, are on the horizontal axis. The color indicates the magnitude of the value at index (*i*,*j*). The value at index (*i*,*j*) of this matrix, $b_{i,j}$, shows the association strength between the *i*-th dimension of the singular vectors and the *j*-th dimension of the plural vectors. Association strengths are highest on the diagonal entries of this matrix, which links every singular with its own plural.

The mean value of the diagonal elements is 0.57 (*SD* = 0.02). Barely any structure is evident elsewhere: the mean value of off-diagonal elements is a mere 9.8 × 10^−5^ (*SD* = 0.017). We can therefore approximate the effect of multiplication with ***B*** with a much simpler operation:

7 $$ \hat{\boldsymbol{Y}} = 0.57 \boldsymbol{X} \boldsymbol{I} + \boldsymbol{\epsilon}, \boldsymbol{\epsilon} \sim {\mathcal{N}}_{300}({-\mathbf{0.001}}, 0.08\boldsymbol{I}), $$

where ***I*** is the identity matrix, is a matrix of 300-dimensional random vectors as row vectors all chosen from the same multivariate normal distribution with mean vector $\mathbf{{-0.001}}$ (a 300-dimensional vector with −0.001 everywhere) and covariance matrix 0.08***I***.^5^ Note that this approximation of ***B*** predicts that the semantic vectors predicted by FRACSS are shorter in length than their singulars: this follows from the multiplication factor 0.57.

How do the FRACSS-predicted vectors compare to the vectors predicted by CosClassAvg? To address this question, we first consider similarity evaluated by means of the angle between vectors and subsequently by means of the Euclidean distance of the corpus-extracted vectors. The median cosine similarity of predicted and target vectors is 0.75 for FRACSS and 0.71 for CosClassAvg (Wilcoxon signed-rank test *W* = 65,105,609.0, *p*≪0.0001 one-tailed, *N* = 11,749). Furthermore, the median cosine similarity between singular vectors and predicted vectors is 0.87 for FRACSS and 0.95 for CosClassAvg (Wilcoxon signed-rank test *W* = 649,018.0, *p*≪0.0001 one-tailed).

When accuracy is evaluated with the cosine similarity measure, the FRACSS plural vectors are close enough to the target plural vectors to capture the plural word correctly as the first nearest neighbor in 1520 cases (Top 1 accuracy: 13%). Similar results are obtained when we use the Euclidean distance measure. The median Euclidean distance to corpus-extracted plural vectors is shorter from predicted vectors for FRACSS at 2.28 in comparison with vectors for CosClassAvg at 2.64 (Wilcoxon signed-rank test *W* = 1,530,725.0, *p*≪0.0001 one-tailed). Conversely, the median Euclidean distance between singular vectors and predicted vectors is 1.67 for FRACSS and 1.04 for CosClassAvg (Wilcoxon signed-rank test *W* = 68,556,852.0, *p*≪0.0001 one-tailed).

**Fig. 13:**
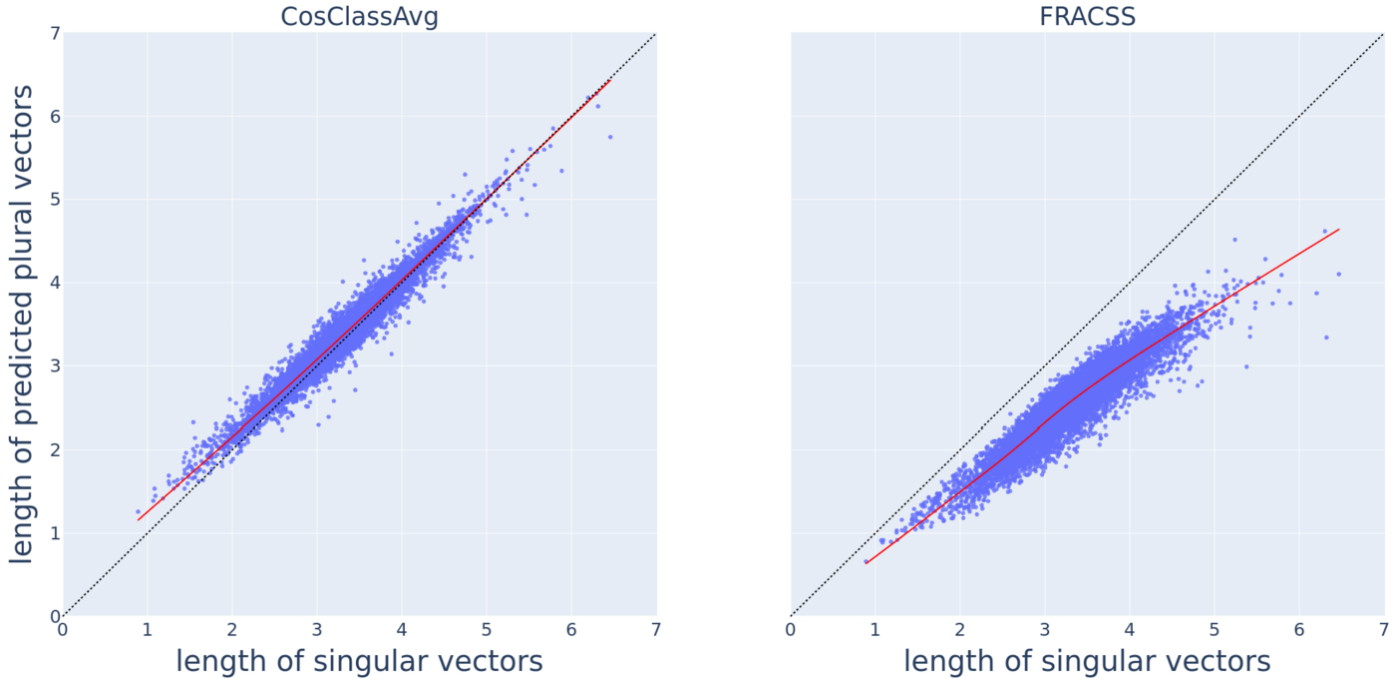
Scatter plots depicting the length of predicted plural vectors, on the vertical axis, versus the length of singular vectors, on the horizontal axis, by CosClassAvg (left panel) and FRACSS (right panel) with the LOcally WEighted Scatterplot Smoothing (LOWESS) trends in solid red lines. The dashed black lines represent the identity line *y* = *x*

Thus far, we have based our evaluation on the angle and distance between vectors. We have seen that FRACSS vectors have smaller angles and shorter distances to plural vectors than CosClassAvg vectors. What about the Euclidean length of the predicted plural vectors? Fig. 13 plots the length of predicted plural vectors against the length of singular vectors, for CosClassAvg (left) and FRACSS (right). For both methods, the length of predicted plural vectors increases with the length of singular vectors, similar to the trend observed in Fig. 6 for the length of corpus-extracted plural and singular vectors. However, there is a striking difference. Most plural vectors predicted by CosClassAvg are longer than their singular vector (74%). By contrast, as anticipated above on the basis of an analysis of the ***B*** matrix, all plural vectors predicted by FRACSS are shorter than their corresponding singular vectors.^6^ However, for the corpus-based actual word2vec vectors, 66% of the plural vectors are longer than the corresponding singular vectors.

We have seen thus far that FRACSS outperforms CosClassAvg when evaluation is based on the angle or distance between vectors, but CosClassAvg outperforms FRACSS when we consider vector lengths. This is perhaps unsurprising as a linear mapping optimizes, for every dimension, the mean squared error and, as a consequence, minimizes the vector length of the estimated plural vectors.

To better understand what the FRACSS mapping does and how it relates to CosClassAvg, let ***S*** denote the matrix with singulars, ***P*** the matrix with plurals, and ***D*** the matrix with shift (difference) vectors. We now decompose the FRACSS mapping matrix ***B*** as follows:

$$ \boldsymbol{B} = \boldsymbol{S}^{-1}\boldsymbol{P} = \boldsymbol{S}^{-1}(\boldsymbol{S}+\boldsymbol{D}) = \boldsymbol{I}+ \boldsymbol{S}^{-1}\boldsymbol{D}. $$

The transformation matrix consists of the identity matrix (which explains why the diagonal elements of ***B*** are so large) to which shift vectors are added that are modified by the inverse of the singular matrix ***S***. If the shift vectors of ***D*** were random, all that ***B*** would do is increment the singular vectors with random values that are weighted by the coordinates of the singular vectors. Without structure in the shift vectors, good generalization to unseen plurals is impossible. For the present data, we have shown that the shift vectors ***D*** are far from random. In fact, by averaging over the shift vectors predicted by the FRACSS mapping, the shift vectors used by CosClassAvg can, to a large extent, be recovered. The FRACSS model offers an advantage as it can also consider the similarities and dissimilarities of words within semantic classes. However, this comes at a cost, namely, the shrinkage of the lengths of the predicted plural vector. The FRACSS mapping is conservative about vector lengths because its ‘cost function’ drives it to keep plural vectors short and close to their singulars.

**Fig. 14:**
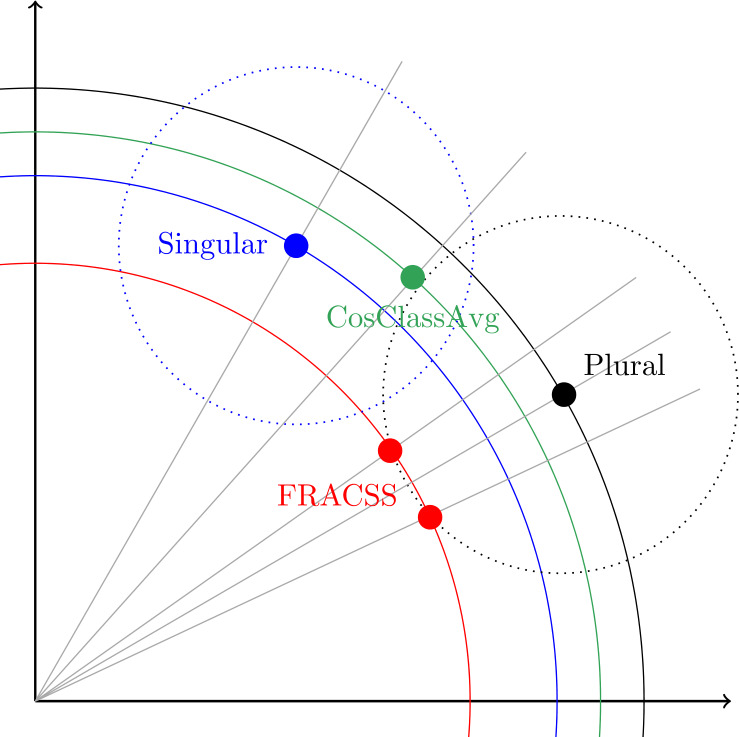
Singular and plural geometry for FRACSS and CosClassAvg in a 2D semantic space

Figure 14 presents the geometry of the solutions offered by CosClassAvg and FRACSS. Plurals tend to have longer vectors than singulars (see Fig. 2). Vector length is depicted by the blue and black circles for singulars and plurals. Plural vectors predicted by CosClassAvg are similar in length to their singular vectors, but the plural vectors predicted by FRACSS are shorter (Fig. 13). Therefore, a CosClassAvg plural vector is located on the blue circle, and a FRACSS vector on the red circle, which has a smaller radius. FRACSS plurals (red) are closer in angle and distance to the observed plural vector compared to CosClassAvg plural vectors (green). CosClassAvg plurals move into the direction of the empirical plural vector, but because the shift vectors are typically too short due to widely scattered outliers (see Fig. 9), the plural vector predicted by CosClassAvg stays too close to the singular. This problem can likely be mitigated by discarding atypical outliers and obtaining improved shift vectors that are at the centroids of the semantic classes. We leave the construction of such vectors to future research. FRACSS vectors, by contrast, are shrunk towards zero, rendering them somewhat more fragile, but they are very well placed in terms of angle and distance from the observed plural. Two possible positions for FRACSS plurals are indicated. The fit is equally good for these examples, which are at the same distance and angle from the observed plural (the sum of their squared errors is exactly the same). Yet, from a linguistic and cognitive perspective, the solution that is closer to the singular seems preferable. Possibly, the problem of predicting plurals with shorter lengths can be avoided by adopting a deep learning algorithm with a cost function that is driven by cosine similarity and distance and penalizes for differences in vector length.

Although this model possesses certain advantages, there are noteworthy aspects to be mindful of before adopting it. Firstly, FRACSS entails higher computational complexity during the phases of training, testing, and updating when compared to the alternative model, CosClassAvg (See appendix for details). Second, there is the risk of overfitting when the training data is small.^7^

### Modelling productivity using DLM

In the preceding sections, we have compared the performance of two methods, CosClassAvg and FRACSS, in terms of the similarity of their predicted plural vectors to word2vec vectors. While both methods exhibit comparable performance, CosClassAvg excels in capturing vector length, whereas FRACSS excels in preserving vector angle in relation to the singular vector. However, when operating in a high-dimensional space, vectors can approach each other in numerous ways. Moreover, beyond their meanings, words also have forms. In what follows, we address the question of how well the forms of plurals are aligned with their meanings, comparing meanings as predicted by 3CosAvg, FRACSS, and CosClassAvg.

To examine the extent to which words’ form space and their semantic space are aligned, we focus on the productivity of the mapping from form to meaning. In English, regular pluralization is considered to be productive, as illustrated by the quote from Lyons (1968) in the introduction. To assess how well a plural’s semantic vector can be predicted from its form, we make use of the Discriminative Lexicon Model (DLM; Baayen et al., 2019; Chuang & Baayen, 2021; Heitmeier et al., 2021; Chuang et al., 2020; Denistia & Baayen, 2022; Heitmeier & Baayen, 2020; Nieder et al., 2023). This is a computational model of the mental lexicon and lexical processing that implements mappings between words’ forms and meanings. In what follows, we use the DLM as a tool for gauging how well words’ form representations map onto their meaning representations when plurals’ semantic vectors are created with 3CosAvg, CosClassAvg, FRACSS, or plain word2vec while keeping words’ form representations the same. Of specific interest is how well the semantic vectors are predicted of plurals that have not been encountered during training. In other words, we are interested which of the four kinds of plurals (3CosAvg, CosClassAVg, FRACSS, and plain word2vec) affords the greatest productivity within the DLM modeling framework.

#### Materials

For training the DLM mappings from form to meaning, we extracted all singular and plural words from the vocabulary dataset introduced in Sect. 3. This subset comprised 8762 English singular and plural words with 9541 unique pronunciations in the NewsScape English Corpus. We constructed training data and test data in such a way that plurals in the test data always had the corresponding singular in the training data. The training data also included plural forms that do not have a corresponding singular in the dataset. Of all plurals with corresponding singulars, 70% were assigned to the training data and 30% to the testing data. This resulted in training data comprising 8507 pronunciations of 7886 words and test data comprising 1034 pronunciations of 1002 words. Table 3 provides further information on the composition of the training and test sets. The training set and the test set contain words spanning across a range of 409 and a range of 296 unique semantic classes, respectively.

**Table 3:** Number of word-form types and tokens in the datasets used for the DL simulations

Dataset	Words	Pronunciations
Training set		
Singular	5073	5511
Plural with seen stem	2253	2412
Plural with unseen stem	560	584
Test set		
Plural with seen stem	1002	1034

Heitmeier et al. (2021) discuss several methods with which numeric representations for word forms can be constructed. In the present study, we make use of numeric form vectors that are based on triphones, i.e., context-sensitive phone units that include information about neighboring segments. For the word *cities*, the triphone cues are #sɪ, sɪt, ɪti, tiz, and iz#, where the # symbol is used to denote word boundaries. For our dataset, there are 6375 unique triphones. A word’s form vector is defined as a vector with a length of 6375 that has values that are either zero or one, depending on whether a triphone is present in a word (1) or not (0). Words’ form vectors can be brought together in a matrix ***C*** with words on rows and triphones on columns (For form vectors derived from the audio signal, see Shafaei-Bajestan et al., 2021). As a result, the matrix with word form vectors ***C*** used for deriving mappings from form to meaning had 8507 rows and 6375 columns.

The form vectors for words are based on the phone transcriptions in the NewsScape English Corpus, which are obtained from the Gentle forced aligner. Gentle’s ASR backend is Kaldi (Povey et al., 2011), which is set up to run with a version of the CMUDict machine-readable pronunciation dictionary (https://github.com/cmusphinx/cmudict), but with information on stress removed. For various words, the dictionary offers pronunciation variants, such as d_B ae_I t_I ah_E and d_B ey_I t_I ah_E for *data*. Here, CMUDict combines ARPABET phone representations with additional information on whether a segment is at the beginning of a word, at an intermediate position, or at the end of a word (B, I, and E respectively).

We note here that the list of pronunciation variants provided by CMUDict is far from complete. For instance, for *ideology*, it provides the transcription /aɪdiɑlʌdʒi/ but not the alternative /ɪdiɑlʌdʒi/. Various reduced forms of function words, as typically found in spoken language, are not represented in the dictionary. For instance, the conjunction *and* is listed with two variants, /ænd/ and /ʌnd/, but forms such as /ʌn/ or even /n/ are not included. As a consequence, the representations we used for words’ forms may not correspond to the exact way in which these words were actually spoken.

#### Evaluating FRACSS and CosClassAvg

For evaluating the advantages and disadvantages of semantic vectors based on CosClassAvg and FRACSS, we set up two semantic matrices, $\boldsymbol{S}_{\text{{CosClassAvg}}}$ and $\boldsymbol{S}_{\text{{FRACSS}}}$ that were based on word2vec. The vectors for singulars were straightforwardly taken from word2vec, but the vectors for plurals were calculated either according to CosClassAvg or according to FRACSS. The two semantic matrices had 8507 rows and 300 columns. We then calculated two 6375 × 300 mappings, $\boldsymbol{F}_{\text{{CosClassAvg}}}$ and $\boldsymbol{F}_{\text{{FRACSS}}}$, by solving the equations

$$\begin{aligned} \boldsymbol{S}_{\text{{CosClassAvg}}} = & \boldsymbol{C}\boldsymbol{F}_{ \text{{CosClassAvg}}} \\ \boldsymbol{S}_{\text{{FRACSS}}} = & \boldsymbol{C}\boldsymbol{F}_{\text{{FRACSS}}}. \end{aligned}$$

With these two mappings, we obtained two sets of predicted semantic vectors for the training data:

$$\begin{aligned} \hat{\boldsymbol{S}}_{\text{{CosClassAvg}}} = & \boldsymbol{C}\boldsymbol{F}_{ \text{{CosClassAvg}}} \\ \hat{\boldsymbol{S}}_{\text{{FRACSS}}} = & \boldsymbol{C}\boldsymbol{F}_{ \text{{FRACSS}}}. \end{aligned}$$

In the above equations, following standard practice in regression modeling, we denote predicted vectors and matrices using the hat notation: the predicted semantic matrix is denoted by $\hat{\boldsymbol{S}}$, and the observed semantic matrix by ***S***. Given the form vectors of the held-out plurals, which we collect as the row vectors of a form matrix $\boldsymbol{C}_{\text{{test}}}$, we also obtain two matrices with predicted plurals:

$$\begin{aligned} \hat{\boldsymbol{S}}_{\text{{CosClassAvg}}, \text{{test}}} = & \boldsymbol{C}{\text{{test}}}\boldsymbol{F}_{\text{{CosClassAvg}}} \\ \hat{\boldsymbol{S}}_{\text{{FRACSS}},\text{{test}} } = & \boldsymbol{C}{ \text{{test}}}\boldsymbol{F}_{\text{{FRACSS}}}. \end{aligned}$$

Prediction accuracy was evaluated by inspecting which gold-standard row vector is closest to the corresponding predicted semantic vector in terms of Pearson’s correlation coefficient. If these vectors belong to the same word (i.e., they have the same row index), prediction is taken to be accurate. In the same way, we can check whether the gold-standard vector is among the top *n* nearest semantic neighbors. Making a prediction for a given test token always involves choosing among 7886 + 1 different semantic vectors—the semantic vectors for the word types in the training set plus the semantic vector for the current test word. Henceforth, we will refer to the DL model with FRACSS vectors as DL-FRACSS and the model with CosClassAvg embeddings as DL-CosClassAvg.

**Fig. 15:**
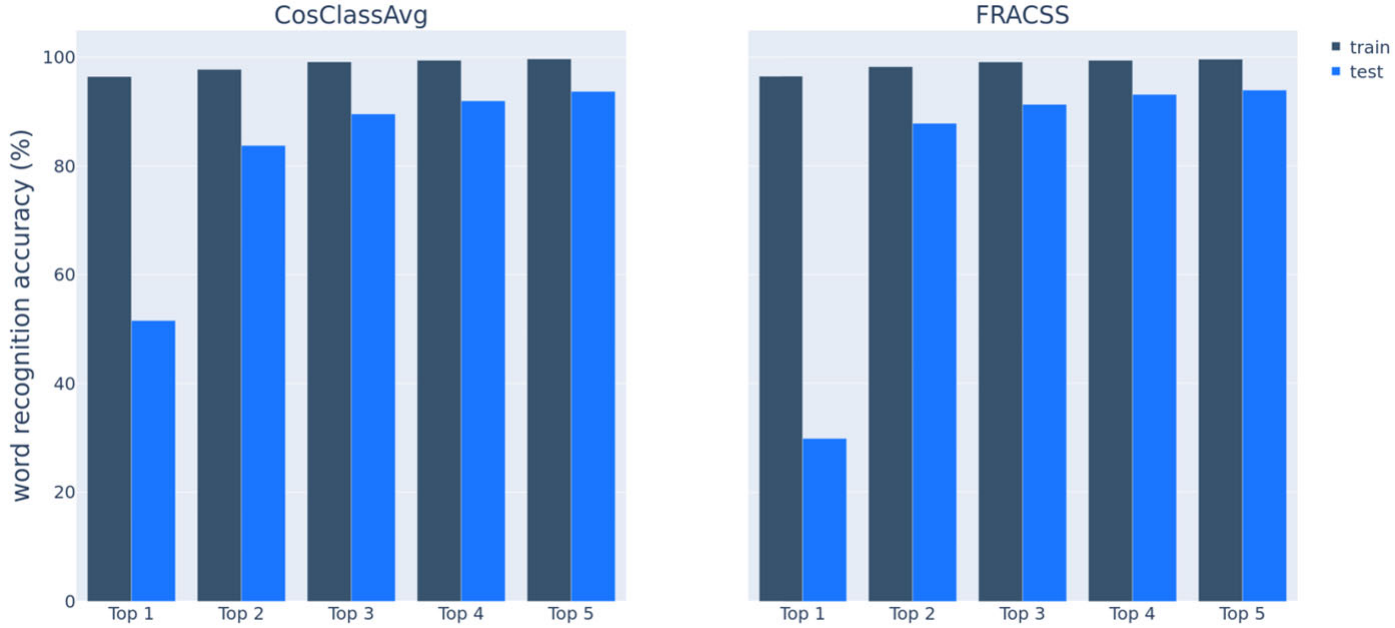
Accuracy of word recognition (%) on the training set (the left column in each pair; *N* = 8507) and on the test set (the right column in each pair; *N* = 1034) by DL-CosClassAvg on the left panel and by DL-FRACSS on the right panel.

Figure 15 presents the top 1 to top 5 accuracies of word recognition evaluated on the training set in dark bars and on the test set in light bars. Recognition accuracy on the training set by both models is 96% for models’ top 1 predictions and increases to almost 100% as we consider the top 2 to top 5 predicted words. With respect to the test data, DL-CosClassAvg outperforms DL-FRACCS by a wide margin in terms of accuracy (top 1),^8^ whereas DL-FRACSS has slightly better performance when the top 2 or top 3 candidates are considered.

Recall that our dataset contains words with multiple pronunciations. The random selection for inclusion in the held-out dataset of seen-stem plural words may result in either having no instances of the plural word in the training set (e.g., both pronunciations recorded for *reports* occur in the test set) or having one pronunciation in the training data and another pronunciation in the test set (e.g., *results* is trained on /ɹɪzʌlts/ and tested on /ɹizʌlts/). DL-CosClassAvg recognizes at least one instance of a word in the test set correctly for 63% of words with multiple pronunciations (*N* = 155). DL-FRACSS performs slightly worse at 46%.

**Fig. 16:**
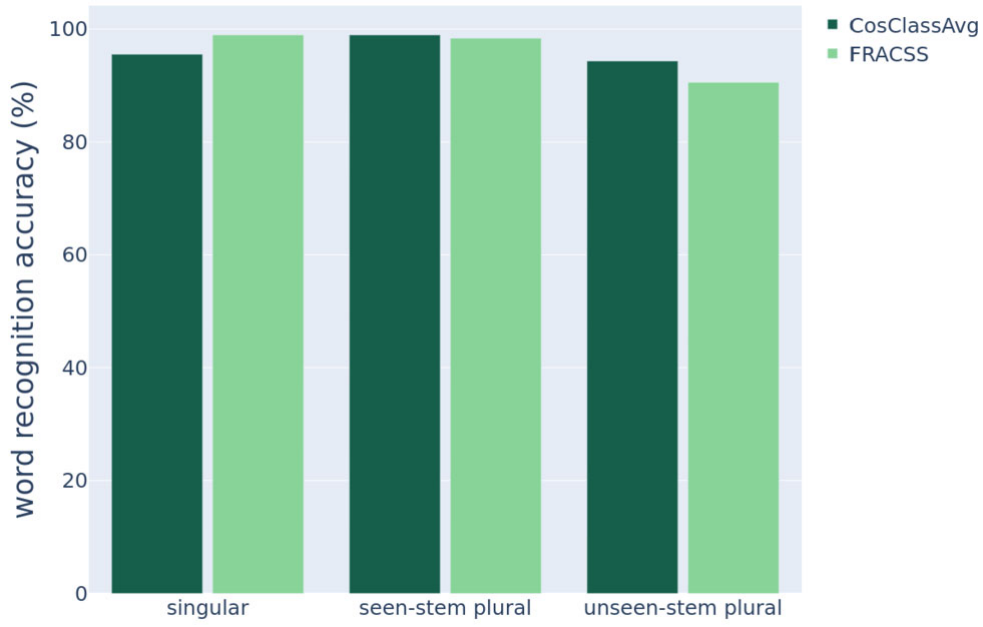
Recognition accuracy on the training set for the singular words, the plural words with a corresponding singular in the training set (seen-stem), and the plural words without a singular in the training set (unseen-stem) by DL-CosClassAvg shown in dark green bars (the left column in each pair) and by DL-FRACSS in light green bars (the right column in each pair)

Figure 16 summarizes model accuracy for the training data. DL-FRACSS is slightly better at recognizing singulars, whereas DL-CosClassAvg performs slightly better for plurals with unseen stems.

**Fig. 17:**
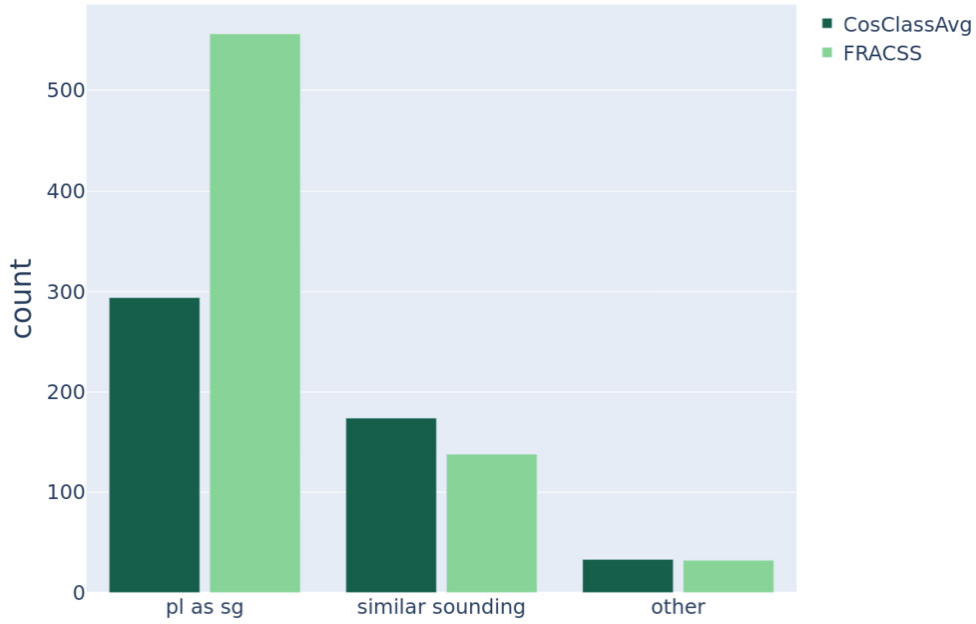
Different types of errors made by the DL-CosClassAvg (the left column in each pair; *N* = 501) and the DL-FRACSS (the right column in each pair; *N* = 726) model on the test set with 1034 tokens

We also examined the kind of errors made by the DL mappings for the words in the test data. Overall, DL-FRACSS makes 726 errors in the evaluation of the test set, and DL-CosClassAvg 501 errors. There are 439 word tokens that both models fail to predict correctly. We distinguished between three types of errors, tabulated in Fig. 17. First, many seen-stem plural words of the test set are recognized as their singular word. FRACSS tends to make more errors of this sort, for which both models frequently get the plural word as their second-best guess, and they always find the plural word among their first four guesses. Highly-ranked competitors tend to be synonyms or semantically related words.

Most of the remaining errors are observed for words with similar forms. To assess this quantitatively, we computed the recall and the overlap indices between the set of target triphones *t* and the set of predicted triphones *p* as follows:

$$\begin{aligned} \text{recall } (t, p) &= \frac{\lvert t \cap p\rvert}{\lvert p\rvert}, \\ \text{overlap } (t, p) &= \frac{\lvert t \cap p\rvert}{\min (\lvert t \rvert , \lvert p\rvert )}. \end{aligned}$$

For example, the word *bribes* is recognized as *tribes* by both models. The predicted and the target word share many form features with a recall and an overlap index of 0.6. We classified words as ‘similar sounding’ when the overlap index was greater than 0.3, and the recall index was greater than 0.2. The remaining words were assigned to the ‘other’ class. The set of words for which a similar-sounding error was made by DL-FRACSS is a subset of that of DL-CosClassAvg. Both models are in error for 33 words in the ‘other’ category, 25 of which are common to the two.

#### Comparison with 3CosAvg and word2vec

We investigated two further sets of semantic vectors for plurals, one based on the 3CosAvg model, and one using the empirical vectors given by word2vec. The mappings are obtained by solving

$$\begin{aligned} \boldsymbol{S}_{\text{{3CosAvg}}} = & \boldsymbol{C}\boldsymbol{F}_{ \text{{3CosAvg}}} \\ \boldsymbol{S}_{\text{{word2vec}}} = & \boldsymbol{C}\boldsymbol{F}_{ \text{{word2vec}}}, \end{aligned}$$

and with these mappings in hand, we obtain the predicted semantic vectors

$$\begin{aligned} \hat{\boldsymbol{S}}_{\text{{3CosAvg}}} = & \boldsymbol{C}\boldsymbol{F}_{ \text{{3CosAvg}}} \\ \hat{\boldsymbol{S}}_{\text{{word2vec}}} = & \boldsymbol{C}\boldsymbol{F}_{ \text{{word2vec}}}. \end{aligned}$$

As plural vectors created with 3CosAvg relate to their singular counterparts in the same way, they exhibit maximum semantic regularity. Conversely, the empirical plurals vectors given by word2vec are the most lexeme-specific, and hence the least regular. In between these two extremes, we find CosClassAvg, which leverages local regularities, and FRACSS, which analyzes the global structure. We, therefore, expect DL-word2vec to be the least productive and DL-3CosAvg to be the most productive, possibly even more productive than DL-CosClassAvg.

Table 4 summarizes the Top 1 accuracy results obtained from evaluating all four mappings on the training and test sets. As expected, the productivity of DL-word2vec is the lowest, at a mere 6%, and the productivity of the most regular semantic vectors is the highest, at 81%. The accuracies for DL-CosClassAVg and DL-FRACSS are intermediate, at 52% and 30%, respectively. Accuracies for the training data are high and very similar for all four models, ranging between 95% and 97%.

**Table 4:**
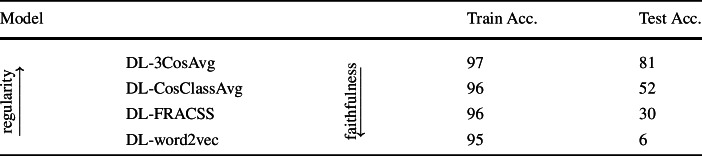
Top 1 accuracy of word recognition for all four DLM mappings evaluated on the training and test sets

These computational experiments point to a trade-off between the regularity built into the semantic vectors on the one hand, and faithfulness to the empirical semantics of the plurals on the other hand. In other words, as we introduce more regularity in the semantic vectors of the plurals, the DLM mapping finds it easier to connect the form space and the semantic space in a way that is productive and makes generalization to novel plurals possible. At the same time, extensively regularizing the pluralization process, as with 3CosAvg, comes at the expense of losing all lexeme-specific information in language usage, as captured by the word2vec algorithm in the corpus-extracted plural vectors. Of the four models that we have studied, the CosClassAvg model appears to provide a reasonable compromise between regularity and productivity on the one hand and faithfulness to the rich semantics of plural words on the other hand.

## General discussion

Using distributional semantics, visualization with t-SNE, and WordNet, we have documented for nearly 15,000 pairs of English singulars and their corresponding plurals that how plural semantics is realized in semantic space varies with the semantic class of the base word. Instead of there being one universal shift from singulars to plurals in distributional space, the direction and length of shift vectors depend on a lexeme’s own semantics. As a consequence, shift vectors for fruits are substantially different from shift vectors for instruments.

The organization of plural shift vectors according to semantic class demonstrates the diverse usage patterns of plural nouns in the English language. A considerable amount of the observed variation arises from differences between concrete and abstract nouns, as revealed by the t-SNE analysis of shift vectors. A majority of abstract concepts appear on the left side of the plot in Fig. 7, whereas concrete concepts appear more in the right-hand side of the graph.

The observed clustering of plural shift vectors by semantic class within the set of concrete nouns likely reflects differences in how plural objects configure in our (culture-specific) constructions of the world. Multiple cars occur in different configurations, which tend to share alignments, as in parking lots or traffic jams. Multiple oranges or multiple cherries occur in very different configurations, typically piled up in boxes or on plates, and bananas occur in hands on banana plants and fruit stands. Our distributional analysis shows that the distinct properties and affordances of these objects that we refer to in the plural are reflected in our language use.

For abstract nouns, similar considerations come into play. Take, for instance, the cognition nouns *encumbrance, hindrance, irritant, impediment*, and *obstacle*, which exhibit very similar shift vectors. Apparently, the usage of plurals such as *encumbrances, hindrances, impediments,* and *obstacles* differs remarkably from plurals of other words such as the state nouns *lymphoma, melanoma, dementia* and *pneumonia* (which also have very similar shift vectors). To us, the plural *dementias* indicates different kinds of dementia, as in the sentences “Dementias that are progressive get worse over time. Types of dementias that worsen and aren’t reversible include …” (Mayo Clinic, dated June 22, 2023). By contrast, *hindrances* and *obstacles* are multiples of states of affairs or dangers to be avoided or overcome. These examples suggest informally that for concrete nouns, their affordances play a crucial role, while for abstract nouns, different construals influence their plural usages.

We proposed the CosClassAvg model to account for the conceptualization of a plural given the singular and its semantic class. According to this model, an empirical plural vector is the sum of four vectors: the vector of the lexeme, the shift vector corresponding to its semantic class, a lexeme-specific vector that captures the lexeme’s unique lexical properties, and an error vector representing measurement noise. We showed that CosClassAvg provides more precise approximations of plural vectors than a model based on a general average shift vector (3CosAvg).^9^

In the subsequent analysis, we proceeded to compare the CosClassAvg model with the FRACSS model (Marelli & Baroni, 2015). Although originally designed for derivation, we explored the applicability of the FRACSS model to plural inflection. Similar to the CosClassAvg model, the FRACSS model takes the semantic vector of the singular as input. However, it employs matrix multiplication instead of vector addition to calculate the semantic vector of the plural. Its use of a single mathematical operation makes FRACSS an ideal candidate for a theory that maintains a single semantic operation of pluralization, as opposed to a more fractionated, semantic-class-based pluralization system seen in CosClassAvg. The results show that the FRACSS model generates plural vectors that are closer to the target plural vectors, both in terms of angle and distance. Nonetheless, the plural vectors produced by FRACSS are comparatively shorter than the target plural vectors.

To better understand the merits of the two models, we further examined the alignment of the FRACSS and CosClassAvg plural vectors with words’ form vectors. This evaluation was conducted using the Discriminative Lexicon Model (Baayen et al., 2019). Two mappings from discrete, numeric form vectors to semantic vectors were established—one utilizing FRACSS to generate plural vectors and the other employing CosClassAvg. For training data, both types of vectors allowed for highly accurate mappings to be established, providing reassurance that neither CosClassAvg nor FRACSS disrupts the mapping between form and meaning.

However, the results from the held-out test data made it evident that plural vectors generated with CosClassAvg yielded substantially superior accuracy in form-to-meaning mappings compared to vectors created with FRACSS.^10^ Since the test data comprised unseen plural words, this finding implies that CosClassAvg exhibits higher productivity for English regular pluralization compared to FRACSS. By further examining an extremely regular method (3CosAvg) and an extremely word-specific method (using the ‘raw’ word2vec embeddings), we were able to show that mapping accuracy increases with the regularity of the plural vectors. Because CosClassAvg implements more regularity in its plural vectors, it is more productive than FRACSS. However, this gain in productivity comes at the expense of losing lexeme-specific information and moving further away from the plural vectors extracted from large corpora.

Does CosClassAvg’s reliance on information about semantic class membership make it more costly to implement than FRACSS? If we consider that the evaluation of computational model complexity must account for all the constructs it utilizes—in our case, the semantic classes of WordNet—then the CosClassAvg model is likely to be more complex than the FRACSS model. However, if we take consulting freely available data as inexpensive lookups, the computational complexity of the FRACSS algorithm becomes higher (see the appendix). Nonetheless, it is at present unclear how complex an end-to-end model would be that does class induction and pluralization jointly. The results obtained with t-SNE and LDA suggest that there is sufficient structure in the embeddings to make such an end-to-end model feasible. In fact, the semantic classes that we took from WordNet are, to a large extent, implicit in word embeddings, as evidenced by the high LDA cross-validation F-scores reported above.

However, it is far from clear to us that such an end-to-end model would actually be desirable, both for linguistic and cognitive reasons. From a linguistic perspective, semantic classes come into play for more than just plural formation. For instance, Booij (1986) pointed out that Dutch agent nouns with the *er* suffix are used to coin nouns falling into a hierarchy of semantic classes (e.g., agent, impersonal agent, and instrument). A theory that is allowed to build on semantic classes for a wide range of morphological phenomena is more parsimonious than a theory that has to induce semantic classes from scratch for each of these phenomena. Furthermore, from a cognitive perspective, knowledge of semantic classes is part of the general knowledge that is acquired as part of our cognitive development and enculturation. Knowing what an orange is includes knowing about the typical configurations in which multiple oranges occur. Language use, as captured by embeddings, reflects this knowledge. In other words, from a cognitive perspective, the knowledge of semantic classes as captured by WordNet, however imperfectly, is a given rather than an explanandum.

In contrast to CosClassAvg, FRACSS exhibits insensitivity to semantic membership as it relies on the global structure between the singular space and the plural space. Although the FRACSS mapping (realized using one matrix operation) seems to suggest that pluralization is a unitary operation represented by one transformation matrix, what this model is actually doing is capturing a wide range of different ways in which plurals are realized, depending in part on the semantic class of their lexemes, but also incorporating lexeme-specific idiosyncracies. From this perspective, FRACSS as applied to plural inflection is very far removed from the plural analogy given by Lyons (1968) that we discussed in the introduction of the present study.

What are the consequences of our findings for the principle of semantic compositionality (Pelletier, 2001) as applied to morphology? According to this principle, the meaning of a plural word is determined by the meaning of the singular and the meaning of the plural suffix, or the meaning that is realized by the rule that creates plurals from singulars. As we have seen, a general shift vector that is the same for all lexemes (as formalized by the 3CosAvg method) can be calculated, affords good productivity, and may have broader value (see Westbury & Hollis, 2019, for experimental evidence concerning clusters of words centered around group averages). However, we have seen that for the English plural, an average shift vector is not very precise. It is possible to take the general shift vector of 3CosAvg be the core of plural compositionality in the sense of Pelletier (1994). In this vein, one would expect different morphosyntactic features to have distinct average shift vectors. However, for English plurals, the common core is a shift vector that is located far outside the cluster of actual shift vectors (see Fig. 5). In fact, the presence of clustering in the shift vectors by semantic class dovetails well with the insight from usage-based grammar and corpus linguistics that individual words, including inflected words, often have their own highly specific usage profiles (see, e.g., Sinclair, 1991). Interestingly, noun pluralization has been characterized as being rather close to derivation: Booij (1996) characterizes it as inherent inflection rather than contextual inflection. In the above formalization of the CosClassAvg model, these insights are expressed at two levels: the level of a word’s semantic class and the level of the individual lexeme.

We have argued that the clustering of plural shift vectors by semantic class reflects differences in affordances of concrete objects and differences in construals for abstract nouns. Following Bresnan et al. (2001), we characterize the conditioning by semantic class of English plural semantics as ‘soft constraints’, which contrast with the ‘hard constraints’ that one finds across many languages.

Some languages split nouns into a group for which plurality marking is relevant and a group for which it is irrelevant. Typically, such splits are made along an animacy hierarchy, from kinship nouns at the highest rank to human nouns to (higher and lower) animate nouns, to inanimate nouns at the lowest rank (Corbett, 2000). In Slave, an Athabaskan language in Northwest Territories, Canada, plural marking occurs optionally only for human nouns and dogs (Rice, 1989). The World Atlas of Language Structures documents 60 other languages that have an optional or obligatory plural marking for human nouns and lack a plural for nouns further down the animacy hierarchy (Haspelmath, 2013).

In Persian, subject-verb agreement in person and number coded on the verb is obligatory for animate plural nouns but optional for inanimate ones (Mahootian, 1997, p. 145). Smith-Stark (1974) reports a similar rule in Georgian. Maori provides a case where number marking is obligatory only for kinship nouns such as *matua* ‘parent’ and *teina* ‘younger sibling’ (Bauer, 1993). In the introduction, we already called attention to the morphosemantics of the Kiowa noun classes. Swahili shows a similar range of semantically motivated classes that determine how a word is inflected for number; see Table 5.

**Table 5:** Exponents for singular and plural for the noun classes of Swahili. Simplified, after Polomé (1967) and https://en.wiktionary.org/wiki/Appendix:Swahili_noun_classes

Class number	Prefix	Typical meaning
1	m-, mw-, mu-	singular: persons
2	wa-, w-	plural: persons (plural counterpart class 1)
3	m-, mw-, mu-	singular: plants
4	mi-, my-	plural: plants (plural counterpart class 3)
5	ji-, j-, Ø-	singular: fruits
6	ma-, m-	plural: fruits (plural counterpart class 5, 9)
7	ki-, ch-	singular: things
8	vi-, vy-	plural: things (plural counterpart class 7)
9	n-, ny-, m-, Ø-	singular: animals, things
10	n-, ny-, m-, Ø-	plural: animals, things (plural counterpart class 9)

Even English has, in a few instances, grammaticalized the diverse ways in which our minds perceive and structure the objects and ideas in the world with which we interact. For English nouns, the distinction between mass and count nouns comes to mind. Additionally, most of present-day English count nouns that never or occasionally take the suffix *-s* in their plural form are names for animals that are hunted (e.g., *duck*, *woodcock*, and *elk*) or fished (*salmon* and *crab*) (see Quirk et al., 1985, for lexemes other than animal names) (see Toupin, 2015, for an extended list of 85 animal nouns).^11^

What are the implications of the present findings for morphological theory? To address this question, we first take the position that the goal of morphological theory is to provide a precise characterization of how stems and exponents combine to form well-formed words given lexemes and bundles of inflectional features. Given this general framework, challenging the conceptual unity of a central inflectional feature such as plurality must be on the wrong track. Current embeddings are noisy; they mix aspects of morphology and syntax (see, e.g., Chuang et al., 2023), and our current understanding of what embeddings actually represent is too limited for the present findings to seriously challenge the usefulness of a central inflectional feature such as plurality. An average shift vector that is far removed from lexeme-specific shift vectors is, from this perspective, at best, a faint echo from language use of a key concept that is part of morphological competence. However, the present findings may possibly shed light on how the hard constraints governing the realization of plurals in a wide range of languages might be grounded in soft constraints, as reported here for English.

But one can also adopt a very different perspective on the task of morphological theory, namely, to model lexical processing in comprehension and speech production. The discriminative lexicon model (DLM Baayen et al., 2019; Chuang & Baayen, 2021; Nieder et al., 2023) provides an error-driven computational framework for setting up and testing mappings between high-dimensional representations of words’ forms and meanings. Results obtained within this framework suggest that there are remarkable isomorphies between high-dimensional representations for words forms and the corresponding high-dimensional representations for their meanings as captured by embeddings (see, e.g., Shafaei-Bajestan et al., 2023; Saito et al., 2022). Within this usage-based approach (see also Heitmeier et al., 2023), the semantic differentiation observed for English plurals is simply a given that any model has to account for. In other words, while acknowledging that current embeddings are far from perfect, the variegated plural semantics that emerge from these embeddings challenge computational modeling of language in use.

One possible way to address this challenge is to replace a single semantic operation for the English plural with a set of nested operations. The simplest, most general model, 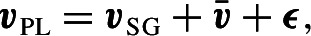
adds the average shift vector $\bar{\boldsymbol{v}}$ to the vector of the singular, while also acknowledging the presence of measurement noise. This model characterizes the interpretation of a plural such as *oak leaves* by a speaker who knows that an oak is some kind of tree, but who does not know what oak leaves look like, nor how they are arranged on the branches of oak trees. The plural semantics are reduced to the average shift vector, which supposedly represents the abstract notion of plurality.

**Fig. 18:**
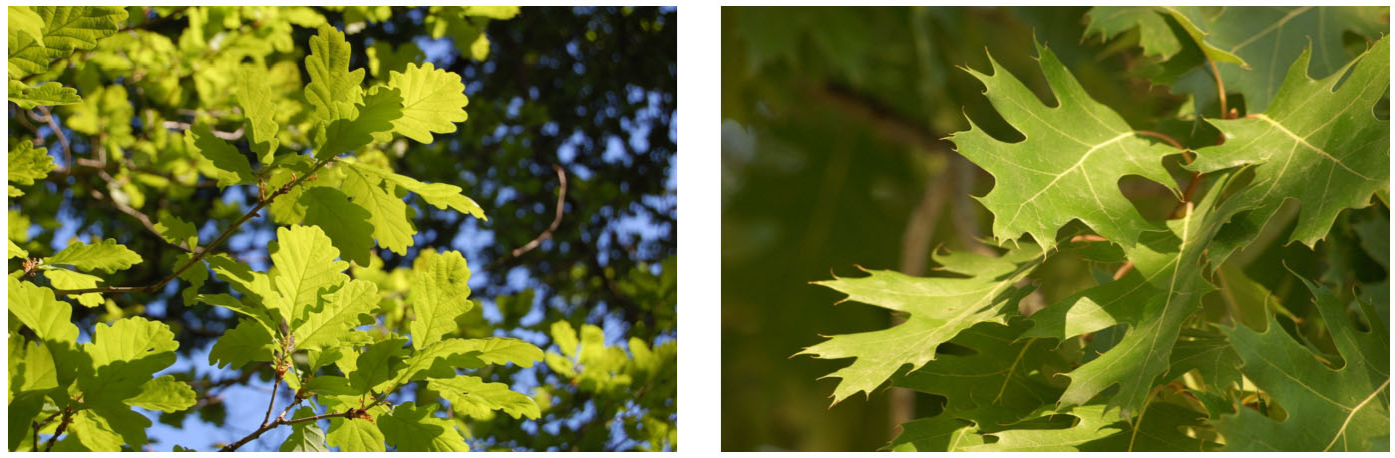
Leaves of the European oak (left) and the American oak (right)

Speakers who are more knowledgeable about oak leaves and their configurations in different kinds of oak trees (see Fig. 18) are able to understand the more specific meaning of *oak leaves* that comes with the semantic class of oak trees. We represent these more specific plural semantics by the vector . This vector specifies the knowledge of shape and configuration that is independent of the abstract vector for plurality. The full plural vector for these speakers is 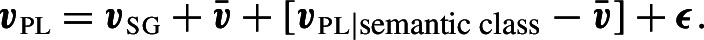

Language users who have specifically the European oak in mind will have an even more specific semantic vector for *oak leaves*, enriched with the knowledge of shape and configuration that distinguishes the European oak from the American oak, represented by $\boldsymbol{\epsilon}_{\text{lexeme}}$: 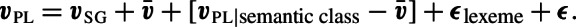
In other words, in this usage-based perspective on plural formation, what a plural means varies with the knowledge of individual language users. Since the word2vec embeddings are trained on huge volumes of data that exceed by far what any individual language user can encounter in real life, the word2vec vectors are more representative of plurals as understood by experts, and less appropriate for language users who are not experts in a given semantic domain.

## Supplementary Information

Below is the link to the electronic supplementary material.

(ZIP 365.2 MB)

## Appendix A: Computational complexity

The computational complexities associated with training, testing, and updating the CosClassAvg and FRACSSS models are summarized in Table A1, considering a corpus containing *t* data points of *n* dimensions. For CosClassAvg, training involves computing average shift vectors, requiring ***O***(*tn*) operations. Semantic category assignment can be performed through an efficient ***O***(1) lookup from freely available resources. Testing the model on a single data point requires vector addition, which takes ***O***(*n*) operations. Updating the model with a new single data point involves re-computing the average shift vector for the corresponding semantic class, taking ***O***(*mn*) operations. Here, *m* represents the number of data points in the semantic class being updated, which is often smaller than *n* (since classes have an average of 28.6 members).

**Table A1:** Computational complexity for CosClassAvg and FRACSS

Method	Training	Testing	Updating
CosClassAvg	***O***(*nt*)	***O***(*n*)	***O***(*mn*)
FRACSS	$\boldsymbol{O}(nt^{2})$	$\boldsymbol{O}(n^{2})$	$\boldsymbol{O}(n^{2})$

The computational complexity of training FRACSS through matrix inversion is $\boldsymbol{O}(n^{3})$. Alternatively, incremental updates can be performed using gradient descent, which has a complexity of $\boldsymbol{O}(nt^{2})$. Testing the FRACSS model on a data point requires a vector-matrix multiplication with $\boldsymbol{O}(n^{2})$. Updating the model can be achieved either by re-computing the entire FRACSS matrix with matrix inversion ($\boldsymbol{O}(n^{3})$) or through a single update using gradient descent that affects the entire mapping ($\boldsymbol{O}(n^{2})$).

## Footnotes

1 Note that the caseless mode is only available for the left3distsim model but not for the slower but usually better bidirectional tagger model.

2 In NLP and Information Retrieval, significant terms provide insights into the content of brief passages in text collections.

3 Following the recommendations of van der Maaten (2021), we searched the t-SNE’s parameter space between possible combinations of *perplexity* (either 10, 15, 20, 25, 30, or 35), *number of iterations* (either 500, 1000, 2000, 3000, or 4000), *random state* (either 1, 12, or 123), and *initialization* method (either random or PCA) for the t-SNE with the lowest Kullback-Leibler divergence. The lowest KL-divergence was obtained with the following setting: perplexity = 35, number of iterations = 4000, early exaggeration = 12, random state = 1, learning rate = ‘auto’, metric =‘euclidean’, and initialization = ‘random’.

4 Cosine similarity between “apple” and “orange” is 0.61 while cosine similarity between “apple” and “banana” is 0.49.

5 For almost all predicted plural and singular vector pairs, the epsilons were normally distributed with an average mean of −0.001 and an average standard deviation of 0.08 (D’Agostino’s $K^{2}$ departure from normality hypothesis test; *p*>0.001 for 99.8% of 11,749 tests).

6 The signed difference between the length of the target plural vectors and the length of the predicted plural vectors is lower for CosClassAvg compared to FRACSS (Wilcoxon signed-rank test *W* = 69,025,375.0, *p*≪0.0001, $\mathrm{MD}_{\text{CosClassAvg}}=0.09$, $\mathrm{MD}_{\text{FRACSS}}=0.89$, *N* = 11,749).

7 This arises due to the large number of parameters it requires, particularly in relation to the dimensions of the input vectors. For instance, in the case of word2vec word embeddings, FRACSS demands 90,000 parameters. While FRACSS tends to perform well when the dataset contains an ample number of data points that surpass the number of parameters, such as the dataset used in the present study, caution is warranted in situations where the data points are significantly fewer than the parameter count. Under such circumstances, FRACSS may exhibit overfitting tendencies and struggle to generalize effectively (Stupak & Baayen, 2023), potentially leading to less desirable outcomes (see, e.g., Ying, 2019, for this widely-recognized problem in machine learning). On the contrary, CosClassAvg is expected to work better as smaller datasets can offer more coherent and possibly fewer semantic classes.

8 The median correlation between the predicted semantic vectors and the target semantic vector is larger for the DL-CosClassAvg model compared to DL-FRACSS (*W* = 214,575.0, *p*<0.0001, $\mathrm{MD}_{\text{DL-CosClassAvg}}=0.78$, $\mathrm{MD}_{\text{DL-FRACSS}}=0.77$, *N* = 1034).

9 Inflection-specific average shift vectors, albeit calculated in a very different way, are also used by the DLM model as laid out in (Baayen et al., 2019), and have the same advantages and disadvantages as vectors obtained with 3CosAvg.

10 Shafaei-Bajestan et al. (2023) show that when mappings are set up between words’ speech signals and their semantic vectors, using linear discriminative learning, plural vectors estimated with CosClassAvg are also better aligned with their audio-derived form vectors compared to FRACSS-based plural vectors.

11 In this study, we kept the stimuli simple and consistent by focusing on regular singular and plural forms. No attempt has been made to zoom in on irregular plurals (e.g., *man* ∼ *men*), zero plurals (*fish* ∼ *fish*), pluralia tantum (*scissors* with no singular variant), singularia tantum (*wealth* with no plural variant), or sense-specific plural formations (*mouse* ∼ *mice* for rodents and *mouse* ∼ *mouses* for computer input devices; Acquaviva, 2008).
